# Regulating Repression: Roles for the Sir4 N-Terminus in Linker DNA Protection and Stabilization of Epigenetic States

**DOI:** 10.1371/journal.pgen.1002727

**Published:** 2012-05-24

**Authors:** Stephanie Kueng, Monika Tsai-Pflugfelder, Mariano Oppikofer, Helder C. Ferreira, Emma Roberts, Chinyen Tsai, Tim-Christoph Roloff, Ragna Sack, Susan M. Gasser

**Affiliations:** 1 Friedrich Miescher Institute for Biomedical Research, Basel, Switzerland; 2 Faculty of Natural Sciences, University of Basel, Basel, Switzerland; University of California San Francisco, United States of America

## Abstract

Silent information regulator proteins Sir2, Sir3, and Sir4 form a heterotrimeric complex that represses transcription at subtelomeric regions and homothallic mating type (*HM*) loci in budding yeast. We have performed a detailed biochemical and genetic analysis of the largest Sir protein, Sir4. The N-terminal half of Sir4 is dispensable for SIR–mediated repression of *HM* loci *in vivo*, except in strains that lack Yku70 or have weak silencer elements. For *HM* silencing in these cells, the C-terminal domain (Sir4C, residues 747–1,358) must be complemented with an N-terminal domain (Sir4N; residues 1–270), expressed either independently or as a fusion with Sir4C. Nonetheless, recombinant Sir4C can form a complex with Sir2 and Sir3 *in vitro*, is catalytically active, and has sedimentation properties similar to a full-length Sir4-containing SIR complex. Sir4C-containing SIR complexes bind nucleosomal arrays and protect linker DNA from nucleolytic digestion, but less effectively than wild-type SIR complexes. Consistently, full-length Sir4 is required for the complete repression of subtelomeric genes. Supporting the notion that the Sir4 N-terminus is a regulatory domain, we find it extensively phosphorylated on cyclin-dependent kinase consensus sites, some being hyperphosphorylated during mitosis. Mutation of two major phosphoacceptor sites (S63 and S84) derepresses natural subtelomeric genes when combined with a serendipitous mutation (P2A), which alone can enhance the stability of either the repressed or active state. The triple mutation confers resistance to rapamycin-induced stress and a loss of subtelomeric repression. We conclude that the Sir4 N-terminus plays two roles in SIR–mediated silencing: it contributes to epigenetic repression by stabilizing the SIR–mediated protection of linker DNA; and, as a target of phosphorylation, it can destabilize silencing in a regulated manner.

## Author Summary

Three Silent Information Regulator (SIR) proteins Sir2, Sir3, and Sir4 are involved in the epigenetic gene silencing of the homothallic mating (*HM*) loci and of telomere-proximal genes in budding yeast. They bind as a heterotrimeric complex to chromatin, repressing the underlying genes. Sir2 has an essential histone deacetylase activity, and Sir3 binds nucleosomes, with a high specificity for unmodified histones. We explored Sir4, whose role had largely remained a mystery. We report here that Sir4 N- and C-terminal domains have distinct functions: The Sir4 C-terminus binds all proteins essential for SIR–mediated silencing and is sufficient to repress *HM* loci, but surprisingly it is not sufficient to efficiently repress at telomeres. The Sir4 N-terminus binds DNA, which strengthens the SIR–chromatin interaction and helps target Sir4 to telomeric loci. In addition the Sir4 N-terminus binds sequence-specific factors that recruit Sir4 to sites of repression. We find that the Sir4 N-terminus is a target of mitotic phosphorylation. Mutation of the phosphoacceptor sites indicates that they help fine-tune subtelomeric repression. We propose therefore that phosphorylation of the Sir4 N-terminal domain modulates epigenetic repression at telomeres in response to cell cycle and/or stress situations.

## Introduction

The eukaryotic genome is organized into euchromatic and heterochromatic domains that generally reflect their potential for gene expression. Chromatin repressed by the Silent information regulator (SIR) complex in the budding yeast *Saccharomyces cerevisiae* shares many key features with heterochromatin in higher eukaryotes. Notably, it has hypoacetylated nucleosomes [1], [2], is less accessible to DNA-binding enzymes than is euchromatin [3]–[5], it replicates late in S phase [6] and is spatially sequestered at the nuclear envelope or near the nucleolus [7]. The genes found within heterochromatin are generally silent, and in complex organisms this gene repression is crucial for the proper development of differentiated tissues and organs [8].

Unlike the situation in other eukaryotes, where histone H3 lysine 9 methylation and its specific ligands mediate repression, heritable transcriptional silencing in *S. cerevisiae* relies on the association of a trimeric SIR complex with unmodified histones (reviewed in [9]–[12]). This heterotrimeric complex contains equimolar amounts of Sir2, Sir3 and Sir4 [13], each of which is essential for the repression of promoters at the homothallic mating type loci, *HMR* and *HML* [14] and in subtelomeric domains [15]. In analogy to centromeric position effect variegation in flies, repression at telomeres has been called telomere position effect, or TPE.

The SIR complex is targeted to the genes it represses by interacting with sequence-specific DNA-binding proteins that bind silencers or telomeric TG repeats. This binding initiates or “nucleates” the formation of silent chromatin on adjacent genes. Repressor activator protein 1 (Rap1; [16]) is a key factor for SIR-mediated repression, because it has high affinity sites both at telomeres and in silencer elements [16], [17]. Furthermore, Rap1 interacts with both Sir3 and Sir4 [18]. *HM* silencer elements contain sites for two further sequence-specific factors, namely Abf1 (ARS-binding factor 1) and ORC (Origin recognition complex) [19], [20]. Abf1 recruits the SIR complex by binding to Sir3 [10], and the largest subunit of ORC, Orc1, enhances SIR recruitment by binding Sir1, an intermediary protein that in turn binds Sir4 [21].

The initial recruitment of Sir4 or Sir3 to telomeric TG-repeats or to silencers, brings in Sir2, a histone deacetylase [22]–[24], which generates high-affinity binding sites for Sir3 by removing acetylation from the histone N-termini of nearby nucleosomes [25]–[27]. Sir3 binds nucleosomes in a manner that is highly sensitive to histone H4 K16 acetylation [28]. The sequential activation of this NAD-dependent histone deacetylase, its generation of high affinity binding sites for Sir3, and their occupancy by the trimeric SIR complex, allow a repressive chromatin structure to propagate along the chromatin fiber [29], [30]. Whereas Sir4 can be recruited to silencer elements independently of Sir2 and Sir3, the spreading of the SIR complex and formation of a silent domain require all three proteins [30], [31]. Mutations that disrupt the interaction between Sir3 and Sir4 compromise repression of the *HM* loci and of genes at telomeres [32], [33].

At 152 kDa, Sir4 is the largest and the least well conserved of the Sir proteins [34]. Its non-globular structure has rendered it refractory to biochemical analysis, except when expressed together with Sir2 [13]. Sir2 and Sir4 form a stable heterodimer, which is mediated by residues 737–839 of Sir4 and a large pocket situated between Sir2's non-conserved N-terminus and its C-terminal catalytic domain (R. Sternglanz and R-M. Xu, personal communication). This tight interaction enhances the de-acetylation activity of Sir2 *in vitro* [13], [35]. Sir4 also interacts with an array of additional factors that are required for efficient repression, leading to its designation as a scaffold for silent chromatin assembly [10], [11]. Importantly, the C-terminal coiled-coil of Sir4 (residues 1257–1358) dimerizes to generate Sir3-binding sites on its outer surface [36], [37], and this interphase is essential for SIR-mediated repression [32]. This coiled-coil domain also binds Yku70 and Rap1 [38]–[41]. Yku70's interaction partner, Yku80, binds two sites within Sir4, one at the Sir4 N-terminus and one in the C-terminal 627 residues [42], [43]. The Ku heterodimer (Yku70/Yku80) not only facilitates SIR recruitment at telomeres, but helps anchor telomeres and silent chromatin at the nuclear envelope, which can enhance the efficiency of SIR-mediated repression [40], [44], [45]. A second, more central domain of Sir4 called PAD (residues 950–1262; partitioning and anchoring domain) also mediates anchorage to the nuclear envelope [42], [46], [47]. The PAD domain of Sir4 binds a nuclear envelope-associated protein called Esc1 (Establishes silent chromatin 1) [47], [48]. Disruption of *ESC1* and *YKU70* or *YKU80* releases telomeres from the nuclear envelope, and selectively de-represses TPE, while repression at *HM* loci remains intact [42], [49], [50].

It is not surprising that the C-terminal half of Sir4 is crucial for silencing, given that it mediates protein-protein interactions with Rap1, Sir2, Sir3, Sir4, Yku70/Yku80 and Esc1. Although we know much less about the functions of the N-terminal part of Sir4, Marshall *et al.* [51] reported that the N-terminus of Sir4 was required for silencing at the *HM* loci. They showed that expression in *trans* of an N-terminal fragment restored mating in the presence of a silencing-deficient C-terminal fragment of Sir4 (the final 45%, starting from about residue 744) [51]. Since then, the first 270 residues of Sir4 (Sir4N) were shown to bind DNA *in vitro* [52] and to interact with three proteins: Sir1 [21], Yku80 [43] and Sif2 [53], a component of the SET3C deacetylase complex [53], [54]. Although Sir4 binding to Sir1 or Yku80 facilitates SIR complex recruitment to *HM* loci and telomeres, neither interaction is essential for SIR-mediated silencing [49], [55], [56]. Thus, it remained mysterious what function the Sir4 N-terminus might have.

Here we have explored the function of the N- and C-terminal domains of Sir4 in silencing at both the *HM* loci and yeast telomeres by means of biochemical and genetic assays. We re-examined the ability of the N- and C-termini to work together in *trans* and found, surprisingly, that a slightly shorter C-terminal fragment (Sir4C; residues 747–1358) than that used by Marshall *et al.* [51], is sufficient to silence *HMR* and *HML* in a *sir4*Δ background. Neither this C-terminal domain nor a fusion protein of Sir4C to the N-terminal 270 residues, however, was sufficient to complement fully a *sir4* deletion for TPE. From this we conclude that the Sir4 N-terminus is dispensable for formation of a repressed chromatin structure, yet it is needed at telomeres or in situations in which SIR complex recruitment is compromised.

We confirmed by biochemical reconstitution assays that recombinant Sir4C is sufficient to form a complex with Sir2 and Sir3 that binds nucleosomal arrays *in vitro* and deacetylates histone H4 K16^ac^. However, Sir4C-containing complexes bind with a four-fold lower affinity and confer less protection of linker DNA from micrococcal nuclease attack. Thus, the DNA binding affinity of Sir4N contributes substantially to the tight association of the SIR complex with chromatin, which becomes important when recruitment is compromised. To see if silencing is regulated through Sir4, we mapped phosphorylation sites within Sir4N *in vivo* and *in vitro*, and found that this domain is a major target for phosphorylation in living cells. Two key phosphoacceptor sites for the cyclin-dependent kinase, serine 63 and serine 84, influence the stability of repression at most telomeres showing TPE. We propose that Sir4N phosphorylation regulates the stability of subtelomeric repression during the cell cycle and possibly in response to environmental stress.

## Results

### Sir4C is sufficient for silencing at intact *HML* and *HMR* loci

**Figure 1:**
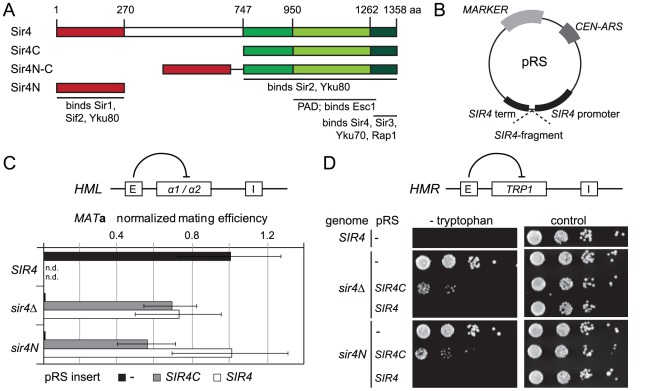
A truncated Sir4C is sufficient for silencing at *HML* and *HMR*. A) Scheme of Sir4 indicating important domains and their interactions. The N-terminal domain of Sir4 (Sir4N) is in red, the C-terminal domains in green (full Sir4C = 747–1358; light green PAD = 950–1262; dark green coiled coil domain = 1262–1358). B) Scheme of plasmids expressing Sir4 constructs. The plasmid's original promoter and terminator were replaced with a 1 kb sequence of the *SIR4* 5′ region and 250 bp of the 3′ region containing the endogenous promoter/terminator information. The same plasmid construct with different markers was used as needed. C) Silencing at *HML* of strains with various Sir4 domains was assayed by quantitative mating to a tester strain (GA858). The endogenous *SIR4* copy was full length (*SIR4*; GA503), a C-terminal deletion (*sir4N*; GA5809) or a complete deletion of Sir4 (*sir4*Δ; GA5822). C-terminal or full length Sir4 was added back on a plasmid. Mating efficiency was normalized to the wild-type strain; data represent mean value ± s.e.m, n.d. undetermined values. D) Plasmids similar to (C), but silencing at *HMR* was assayed using a *TRP1* reporter (GA484, GA6072, GA5886). Serial dilutions of transformed strains were grown on control plates selecting for the plasmid only or on plates selecting for the plasmid and growth without tryptophan (monitoring repression of *TRP1*).

To examine the function of the N-terminus of Sir4, we first repeated the assay of Marshall *et al.* [51] in which N- and C-terminal fragments of Sir4 were expressed in *trans* and scored for the restoration of silencing at *HML* in a *sir4*Δ background. We created strains with either a full deletion of *SIR4* (*sir4*Δ) or with a partial deletion of the endogenous *SIR4* locus (*sir4N*), such that only its N-terminal 270 amino acids were expressed. We then expressed full-length Sir4 or various C-terminal fragments of the protein from CEN-ARS plasmids (pRS) carrying the full *SIR4* promoter and terminator (Figure 1A, 1B). Because overexpression of either full-length protein or fragments of Sir4 derepress gene silencing [53], we chose conditions that reproduced as closely as possible the endogenous Sir4 protein levels (Figure S1B and data not shown). Quantitative mating assays can be used to determine the degree of repression at *HML*, because mating is compromised by coincident expression of **a** and α mating type information. In contrast to the findings of Marshall *et al.* [51], expression of a C-terminal fragment of Sir4 (Sir4C, residues 747–1358) alone was sufficient to repress *HML*, as indicated by the restoration of mating in a *MAT* **a** *sir4*Δ strain (Figure 1C, Table 1). Consistently, expression of Sir4C also repressed a *TRP1* reporter inserted at *HMR* in both the *sir4*Δ and *sir4N* backgrounds (Figure 1D, Table 1).

**Table 1:** Summary of silencing phenotypes.

Genome	Sir4 CEN plasmid	HML (mating)	HML (mRNA)	HMR (*TRP1*)	HMR::ΔA (*TRP1*)	HMR:: ΔB (*TRP1*)	Tel7L (*URA3*)	Tel7L (*URA3* mRNA)	Tel5R (*ADE2*)	Tel6R (mRNA)	Tel9R (mRNA)
*sir4*Δ	Sir4	**+++**	**+++**	**+++**	**+++**	**+++**	**+++**	**+++**	**+++**	**+++**	**+++**
*sir4*Δ	Sir4C	**+++**	**++**	**++**	**+**	**−**	**−**	**+**	**−**	**−**	**−**
*sir4*Δ	Sir4N-C	**++**	**++**	**+++**	**+++**	**+++**	**−**	**+++**	**−**	**−**	**+**
*sir4*Δ	Sir4^731–1358^	**−**	n.d.	**−**	n.d.	n.d.	**−**	n.d.	**−**	n.d.	n.d.
*sir4N*	Sir4	**+++**	n.d.	**+++**	**+++**	**+++**	**+++**	n.d.	**+++**	n.d.	n.d.
*sir4N*	Sir4C	**+++**	n.d.	**+++**	**+**	**−**	**−**	n.d.	**−**	n.d.	n.d.

Summary of silencing phenotypes of Sir4 C-terminal constructs on different reporters and backgrounds. Qualitative silencing strength is given (+++: same level as full length *SIR4*, ++intermediate silencing, +weak silencing, −no silencing (same as *sir4*Δ), n.d. undetermined value).

In trying to explain the discrepancy between our findings and those of Marshall and colleagues, we noticed that they had used a galactose-inducible Sir4 C-terminal fragment that was a few amino acids longer than ours, and co-expressed as well a slightly longer N-terminal fragment than we used [51]. Intriguingly, our analysis of a longer C-terminal fragment (residues 731–1358; Sir4^731–1358^), showed that it failed to repress an *HMR*::*TRP1* reporter, either alone (in a *sir4*Δ background) or when expressed with Sir4N (in a *sir4N* background; Figure S1A). Immunoblotting showed that steady-state levels of the Sir4^731–1358^ fragment were much lower than of those of the shorter Sir4C (Figure S1B). The instability of the Sir4^731–1358^ fragment would explain its inability to repress *HMR*; indeed, it is likely that the fragment used by Marshall and colleagues was also unstable, and therefore did not silence on its own. We tried also expressing a longer (330 residue) N-terminal fragment with both long and short Sir4C fragments, but observed no differences in the mating assay compared to the shorter Sir4N fragment (data not shown). Our results suggest that a stable 611-residue C-terminal fragment of Sir4 is sufficient to repress both *HM* loci.

### The N-terminus of Sir4 contributes to repression at *HM* loci with incomplete silencers

**Figure 2:**
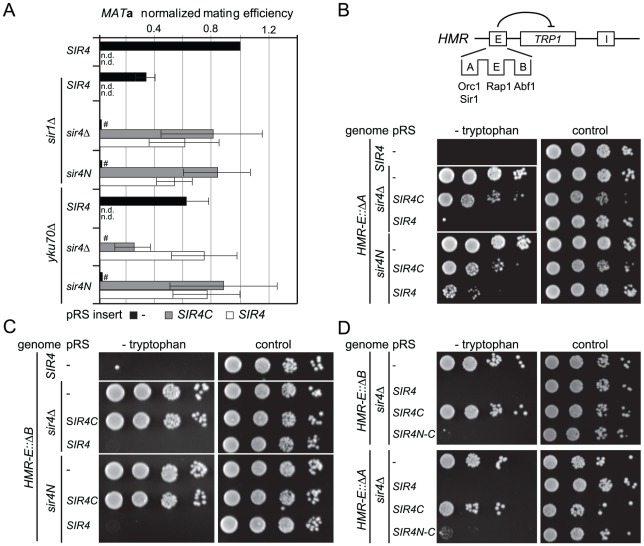
Sir4C is not sufficient for silencing at compromised *HM* loci. A) Quantitative mating assays were performed as in Figure 1C with strains additionally carrying full deletions of *YKU70* or *SIR1* (GA6069, GA6070, GA6071, GA6062, GA6063, GA6064). Mating was normalized to wild-type cells and at least three independent experiments were quantified; data represent mean value ± s.e.m. # indicates values below 10^−3^, n.d. undetermined values. B, C, D) Testing silencing of compromised *HMR*: full *sir4* deletion or endogenous *sir4N* were complemented with *SIR4*, *SIR4C* or a *SIR4N-C* fusion in strains carrying a *TRP1* reporter at *HMR*. The *HMR-E* silencer carried a deletion of either the B (Abf1 binding; GA485, GA6888, GA6899) or A (Orc1-Sir1 binding; GA486, GA6890, GA6891) site. Dilution series for repression were performed as in Figure 1D.

To date, the N-terminus of Sir4 was implicated in recruiting the SIR complex to silencers or to telomeres through its affinity for Sir1 or Yku80, respectively [21], [40], [41], [56], [57]. The interactions that recruit the SIR complexes to silencers are, however, redundant [20]. Therefore, we next tested the impact of Sir4N on silencing under conditions of compromised recruitment, that is, in strains lacking either Sir1 or Yku70 which eliminates Yku80 function as well (Figure 2). The expression of Sir4C in a *sir1*Δ strain could still restore silencing of *HML*, either in the absence (*sir4*Δ) or the presence of Sir4N (*sir4N*, Figure 2A). Consistent with it being a direct binding partner of Sir1, Sir4N expression did not enhance silencing in the *sir1*Δ background (Figure 2A). On the other hand, in the *sir4*Δ *yku70*Δ background, Sir4C only supported mating at 30% of wild-type levels. In this case, mating efficiency was indeed enhanced by co-expression of Sir4N (compare *SIR4C* in *sir4*Δ and *sir4N*, Figure 2A). This confirms that the N- and C-termini of Sir4 can complement in *trans* at the *HML* locus, as reported by Marshall *et al.* (1987), although in our hands, this is true only in *yku70*Δ cells.

To assess more directly whether Sir4N compensates for the absence of other recruitment sites at silencers, we used a *HMR::TRP1* reporter strain that lacks either the A sequence (ORC–Sir1-binding site) or the B sequence (Abf1-binding site) within the E silencer (Figure 2B) [19], [20]. When we deleted the ORC–Sir1-binding element (*HMR-E*ΔA), Sir4C was no longer sufficient to repress the reporter gene at *HMR* (Figure 2B, Table 1). Similarly, in the absence of the Abf1-binding site (*HMR-E*ΔB, Figure 2C, Table 1), Sir4C did not restore silencing, either with or without the Sir4N fragment. Thus, Sir4C is not sufficient for silencing at an *HMR* locus in which the silencers are weakened by deletion of a binding site for one of the recruitment factors.

The co-expression of Sir4C and Sir4N in *trans* did not enhance silencing at compromised silencers, as they did in the *yku70* mutant (Figure 2A–2C). This may be explained if Sir4N interacts only weakly with the SIR complex. To test this possibility, we tethered the Sir4N and Sir4C domains with a short linker peptide, to form a stable fusion protein (Sir4N–C; Figure 1A). Importantly, when expressed in a *sir4*Δ background, Sir4N-C repressed the reporter gene as effectively as full-length Sir4 at the silencer-compromised *HMR* loci (*HMR-E*ΔA and *HMR-E*ΔB; Figure 2D, Table 1). The strains expressing Sir4N-C were also competent for mating (Figure S2A), albeit with lower efficiency than cells expressing Sir4C alone, possibly due to an altered growth rate (see legend, Figure S2). These data confirm a role for the N-terminus of Sir4 in silencing the *HM* loci when the binding of recruitment factors is compromised. Indeed, at *HMR* with weakened silencers, the expression of a Sir4 N-terminal fragment along with Sir4C allows repression, whereas Sir4C alone does not.

### Linking Sir4N to Sir4C increases but does not fully restore telomeric silencing

**Figure 3:**
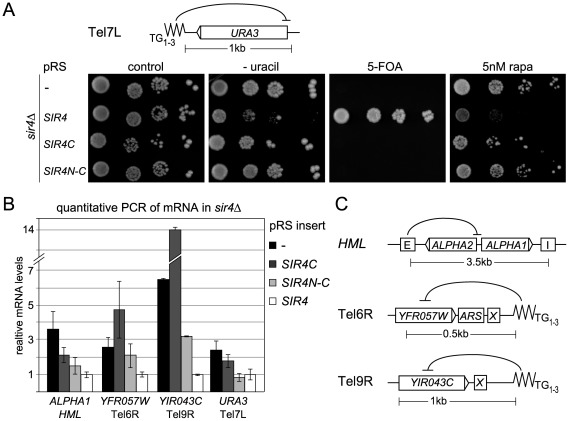
Sir4C is not sufficient for silencing at telomeres. A) Telomeric silencing was monitored by a Tel7L::*URA3* reporter gene (GA503, GA5809, GA5822) expressing the indicated proteins from pRS, including the SIR4N-C fusion. Growth on plates containing 5 nM rapamycin (rapa) was also monitored. B) Relative mRNA levels of three different subtelomeric genes and *HML-ALPHA1* were measured using QPCR. Bars represent averages of biological triplicates, data represent mean value ± s.e.m. C) Scheme of the *HM* loci and telomeres analyzed, indicating additional recruiting elements and distances of promoters from nucleating elements.

To test whether silencing at telomeres requires the N-terminus of Sir4, we monitored expression of a *URA3* reporter gene at telomere 7L (Tel7L::*URA3*) [4] by assaying growth in the absence of uracil in a strain that lacks Ppr1, the transcription factor responsible for inducing *URA3* in auxotrophic conditions [58]. In contrast to repression at the *HM* loci, telomeric silencing could not be established by expressing Sir4C (Figure 3A, Table 1) nor by co-expressing Sir4C with Sir4N in *trans* (Figure S2B, Table 1). This was true not only for *URA3* expression at Tel7L, but also for the *ADE2* reporter gene expression at Tel5R (Figure S2C, Table 1). We also monitored Tel7L::*URA3* repression by counter-selecting with the drug 5-FOA, with similar results (Figure 3A).

Given that repression at *HMR* with weakened silencers was enhanced by expression of a Sir4N-C fusion, we tested the effect of this hybrid on TPE. Surprisingly, expression of the Sir4N-C fusion in a *sir4*Δ strain failed to repress either Tel7L::*URA3* or Tel5R::*ADE2* reporters in the standard drop assay (Figure 3A, Figure S2C, Table 1). We also assayed silencing by measuring mRNA levels of subtelomeric genes from telomeres 6R and 9R by quantitative PCR (QPCR; Figure 3C). Both genes were derepressed in cells expressing only Sir4C or Sir4N-C. For Tel9R we observed partial repression by Sir4N–C compared to *sir4*Δ (Figure 3B, Table 1). Intriguingly, the levels of the *HML*α1 gene were only partial reduced when Sir4C was expressed, whereas expression of Sir4N–C conferred repression to near-background levels (Figure 3B). A similar effect was observed at Tel7L::*URA3* when *URA3* was transcribed at basal levels (i.e. growth in the presence of uracil and in the absence of Ppr1; Figure 3B). In this case, Sir4N–C repressed transcription to background levels, unlike in the drop assay in the absence of uracil (Figure 3A), which strongly induces transcription from the *URA3* promoter.

Resistance to rapamycin is a sensitive means to monitor native telomeric silencing, as growth on the drug requires expression of multiple stress genes located near telomeres, which are normally silenced by the SIR complex [59]. We therefore monitored the level of stress gene expression by scoring for resistance to rapamycin in Sir4-, Sir4C- and Sir4N–C-expressing cells. Whereas *SIR4^+^* cells fail to grow on rapamycin, intriguingly, both Sir4C- and Sir4N–C-expressing cells behaved like *sir4*Δ when grown in the presence of rapamycin (Figure 3A). This argues that neither Sir4C nor Sir4N–C can prevent the induction of natural subtelomeric genes by stressful conditions (Figure 3A), suggesting that full-length Sir4 is needed for native subtelomeric repression, although not at *HM* loci.

Transcriptional repression generally correlates with the binding of Sir proteins throughout the silent domain, and we therefore tested the binding of Sir4 to *HML* and telomeres by chromatin immunoprecipitation (ChIP). We detected a clear enrichment of Sir4, Sir4C and Sir4N-C at *HML*-E and *HML*-α1 (Figure S2F). Consistent with the silencing assays, on the other hand, only full length Sir4 was strongly enriched at telomeres (Figure S2F). This confirms that full-length and truncated Sir4 proteins are bound at the sites that are silenced robustly, and shows again that Sir4C is not sufficient for binding in subtelomeric domains.

To see if Sir4C would be sufficient for silencing at telomeres if we enhanced SIR recruitment by Rap1, we monitored TPE in the absence of the Rap1-interacting factor 1 (Rif1), which competes for Sir3 and Sir4 recruitment by the Rap1 C-terminus [60]. Whereas deletion of *RIF1* increased telomere length and SIR recruitment, leading to enhanced silencing [18], [41], it did not increase Sir4C- or Sir4N–C-mediated repression at Tel7L or Tel5R::*ADE2* (Figure S2D, S2E; compare to Figure 3A). Taken together, these data indicate that Sir4C is insufficient for TPE, and that Sir4N can contribute weakly to improve repression at native telomeric genes and reporters, yet only full-length Sir4 supports robust TPE.

### Sir4C can form a stable and active SIR complex

**Figure 4:**
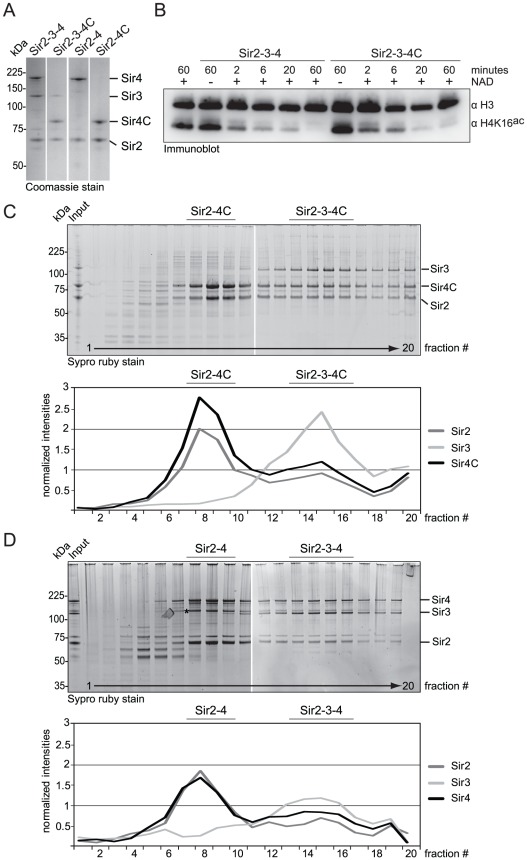
Sir4C can form a stable and active SIR complex in a recombinant system. A) SIR complexes as indicated were purified form co-infected insect cells. 1 µg of each complex was run on a SDS-PAGE and visualized by Coomassie staining. B) Purified Sir2–Sir3–Sir4 and Sir2–Sir3–Sir4C complex were incubated with histone octamers acetylated at H4K16 with or without the essential cofactor NAD. The deacetylation reaction was stopped after various time points by the addition of sample buffer and monitored by immuno blotting for H4K16^ac^ and H3, for equal loading. C, D) Sir2–Sir3–Sir4 or Sir2–Sir3–Sir4C complexes were analyzed by density gradient sedimentation. Fractions were run on 4–12% NuPAGEs Novex Bis-Tris Gels and stained with Sypro Ruby dye. Intensities of Sir2, Sir3 and Sir4 full length proteins were quantified (QuantityONE) and plotted in line graphs. The asterisk in D) indicates a Sir4 degradation band that runs very closely to Sir3.

Because Sir4C can silence *HM* loci, we asked whether Sir4C forms a stable complex with Sir2 and Sir3. To test this, we co-expressed Sir4C with Sir2 and Sir3 in baculovirus-infected insect cells. Using conditions identical to those used to purify the full-length Sir2–Sir3–Sir4 complex, we were able to purify a SIR complex containing Sir4C (Figure 4A, 4C; [13], [52]). Upon glycerol density gradient sedimentation the complex migrated in two distinct complexes: one containing Sir2, Sir3 and Sir4C and the other containing only Sir2 and Sir4C, exactly like the complex with full-length Sir4 (Figure 4C, 4D; [13]). We conclude that Sir4C is sufficient to form a complex similar to the wild-type SIR complex, when expressed in insect cells.

To confirm that the Sir4 N-terminus is dispensable for the deacetylation activity of the SIR complex, we incubated recombinant protein complexes of either full-length Sir2–Sir3–Sir4 or truncated Sir2–Sir3–Sir4C with histone octamers that were fully acetylated on histone H4K16. We assayed H4K16^ac^ deacetylation over time by Western blotting [28], and found that the two complexes had similar deacetylation activities (Figure 4B). We conclude that Sir4C forms a stable and active SIR complex, consistent with its ability to confer *HM* repression.

### Sir4N promotes high-affinity binding to chromatin and linker DNA protection

**Figure 5:**
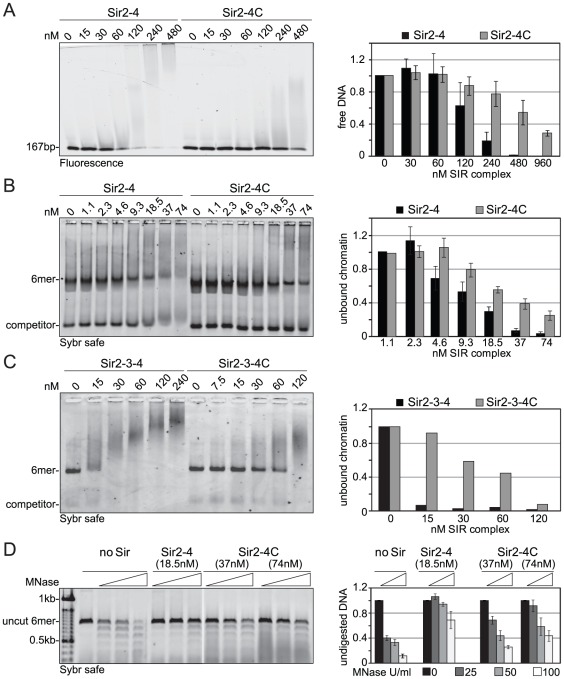
Sir4C has reduced affinity for DNA and chromatin and protects linker DNA less from MNase attack. A) Increasing amounts of Sir2–Sir4 or Sir2–Sir4C complexes were titrated into a fixed amount of 167 bp 601-Widom Cy5-labeled DNA. Samples were separated by native agarose gel electrophoresis and visualized by Cy5 fluorescence. Binding in three independent experiments was quantified by measuring the disappearance of the unbound DNA and normalized to input; data represent mean value ± s.e.m. B) Sir2–Sir4 and Sir2–Sir4C complexes were titrated into constant amounts of 6 mer arrays of unmodified nucleosomes. Samples were analyzed as (A); chromatin was visualized by SybrSafe staining. C) Sir2–Sir3–Sir4 and Sir2–Sir3–Sir4C complexes were titrated into constant amounts of 6 mer arrays of unmodified nucleosomes as in (B), with only one experiment analyzed. D) Indicated concentrations of Sir2–Sir4 or Sir2–Sir4C were bound to chromatin as in (B) and incubated with increasing amounts of MNase for 10 min on ice prior to deproteinization. DNA was analyzed by agarose gel electrophoresis and SybrSafe staining. The amount of full length 6 mer DNA was quantified to monitor degree of digestion. Data from at least three experiments are represented as mean value ± s.e.m.

To explain the contributions of the Sir4 N-terminus for repression in biochemical terms, we examined the contribution of Sir4N to SIR complex loading onto nucleosomal arrays *in vitro*. In a previous study, we showed that recombinant Sir4N has considerable non-specific affinity for DNA [52]. To test whether this contributes to the affinity of the SIR complex for chromatin, we first compared the DNA-binding properties of the Sir2–Sir4 and Sir2–Sir4C complexes. Increasing amounts of each complex were titrated into a constant amount of a high-affinity histone octamer-binding sequence (Widom 601; [61]). By using the binary Sir2–Sir4 complex rather than ternary complexes with Sir3, we could avoid contributions of Sir3 to DNA binding [26]. SIR complex association with DNA leads to the appearance of higher molecular weight species after native gel electrophoresis, and the disappearance of unbound DNA. We quantified the disappearance of the unbound DNA as a function of Sir2–Sir4 complex concentration. This showed that the truncated Sir2–Sir4C complex has about four–fold lower affinity than the full-length Sir2–Sir4 complex for naked DNA (Figure 5A).

We next examined the contribution of the Sir4 N-terminus to nucleosome binding, by titrating the complexes onto hexameric nucleosomal arrays assembled *in vitro*, as previously described [52]. Again, by quantifying the disappearance of unbound nucleosomes, we found that the Sir2–Sir4C complex has roughly two-fold lower affinity for chromatin than the full-length Sir2–Sir4 complex (Figure 5B). This effect was even more pronounced when we compared the binding of holo-SIR complex with that of the Sir2–Sir3–Sir4C complex. The complex carrying the truncated Sir4C had a much lower affinity for nucleosomal arrays than that containing full-length Sir4 (Figure 5C), possibly because Sir3 sterically masks part of Sir4C's chromatin-binding surface [27], [33].

Since the SIR complex is known to protect nucleosomal linker DNA from micrococcal nuclease (MNase) attack [28], [52], we examined the contribution of Sir4N to linker DNA protection. Importantly, we used two- to four-fold more of the truncated Sir2–Sir4C complex than of wild-type Sir2–Sir4 complex, to ensure that equal amounts of chromatin-SIR complex were formed (Figure 5B). The Sir2–Sir4C complex showed less linker DNA protection than the full-length Sir2–Sir4 complex (Figure 5D), despite the fact that equal fractions of nucleosomes were bound in each reaction. These data suggest that the affinity of Sir4N for DNA promotes a tighter binding of SIR complexes to chromatin, thereby enhancing linker DNA protection. This attributes a function to the Sir4 N-terminus beyond recruitment by Sir1 or Yku80.

### Truncated Sir4 mediates formation of Sir3 foci independently of silencing

**Figure 6:**
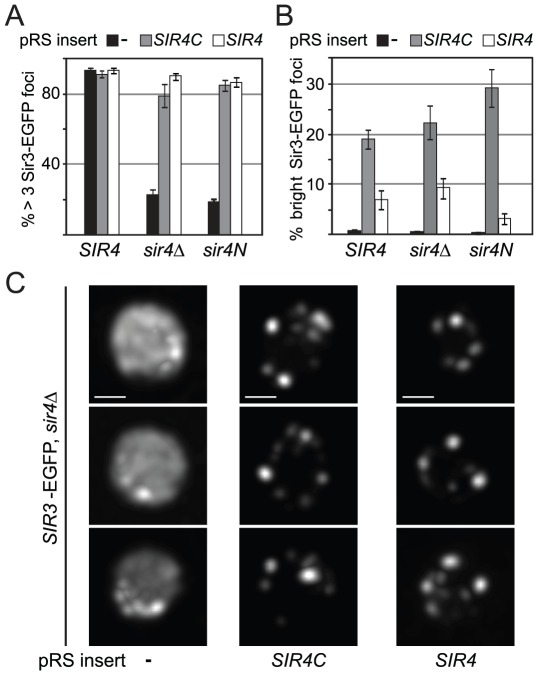
Sir4C supports Sir3 focus formation *in vivo*. A, B) Sir3-EGFP foci were monitored in logarithmically growing cultures using live microscopy. Sir3-EGFP was tagged at its endogenous locus and the strains carried the indicated forms of *SIR4* (GA3128, GA6287, GA6288). Full-length Sir4 or Sir4C were added back on plasmids. Images were quantified by counting cells having >3 Sir3 foci at a low signal threshold or >1 Sir3 focus at a high threshold of equally treated images (n>240 cells/sample; >2 independent experiments; data represent mean value ± s.e.m). C) Single focal planes of deconvolved images of Sir3-EGFP as above; size bar 1 µm.

Silencing at telomeres is sensitive to the anchorage and clustering of the telomeres at the nuclear envelope [44], [45], [50]. Since Sir4C can restore silencing at *HM* loci but not at telomeres, we wondered whether Sir3 focus formation, as an indication of telomere clustering, might depend on Sir4N. To test this hypothesis, we expressed a Sir3–EGFP fusion protein in yeast cells in which the endogenous *SIR4* gene was either deleted (*sir4*Δ) or truncated (*sir4N*), and either *SIR4* or *SIR4C* was expressed from a CEN-ARS plasmid. Similar to a strain without tagged Sir3 (Figure 3A, S2B), both Sir4C and full-length Sir4 restored mating-type repression in the *SIR3*–*EGFP* background, but only full-length Sir4 was able to restore TPE fully (Figure S3). Although Sir4C-expressing cells are competent to mate, they grow more slowly (Figure 2C) and occasionally had larger and obviously distorted nuclei compared to wild-type cells. We imaged Sir3-EGFP in living cells and found that Sir3–EGFP foci formed when either Sir4 or the truncated Sir4C protein was expressed, as determined by counting the number of cells containing at least three Sir3–EGFP foci (Figure 6A, 6C). In about 20% of the Sir4C-expressing cells, we observed one to three very intense Sir3 foci in addition to the small telomere clusters, whether or not the endogenous *SIR4* gene was present (Figure 6B). We conclude that the N-terminus of Sir4 is not necessary for the formation of Sir protein clusters, and thus that large Sir3–EGFP foci can form in the absence of TPE. The dissociation of Sir3 foci from TPE confirms a recent report showing that non-perinuclear Sir3 clusters can form in cells unable to support SIR-mediated repression [62].

### The Sir4 N-terminal domain is its major site of phosphorylation in G2/M-phase cells

SIR complex binding at telomeres appears to be modulated in response to the physiological state of the cells. For instance, Sir proteins are released from telomeres both in mitotic cells [63], [64], and in response to genotoxic stress [65], [66]. Indeed, activation of the DNA damage checkpoint affects TPE, but not *HM* repression, much like the deletion of Sir4N. Moreover, subtelomeric domains contain a number of genes that are regulated in response to nutrient stress [67], [68] by a kinase cascade that targets, among other things, Sir3 [59]. Finally Sir4N harbors many potential phosphoacceptor sites, and whole phosphoproteome studies suggested that Sir4 is modified in a manner that fluctuates with the activity of the cell-cycle regulated cyclin-dependent kinase (CDK; [69], [70]). Thus it was proposed that the N-terminal half of Sir4 might act as a phosphorylation-dependent regulatory domain [34].

**Figure 7:**
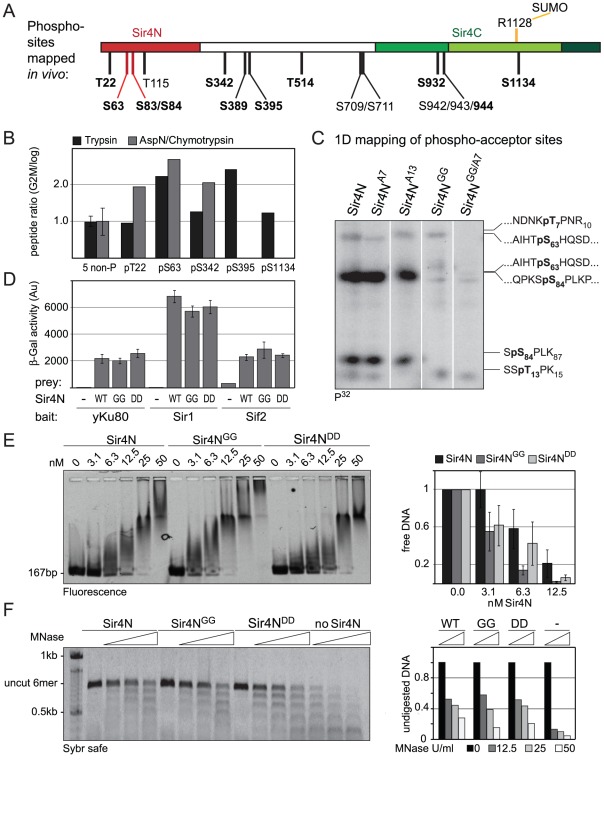
Sir4N is the major site of phosphorylation. A) Scheme of Sir4 as in Figure 1A, indicating identified phosphoacceptor sites of full-length Sir4. Serine 63 and 84 mutated in subsequent experiments are indicated in red. Sites having the [S/T*]-P consensus are in bold. B) Relative quantification of the enrichment of Sir4 phosphopeptides in G2/M over cycling cells by LC-MS of a trypsin and of a combined AspN/chymotrypsin digest of a Sir4-IP experiment. For each digest the extracted ion chromatograms were integrated and the ratios of peptides detected in G2/M versus log growing cells were calculated for five phosphorylated as well as five non-phosphorylated Sir4 peptides. The ratio average of the five non-phosphorylated peptides of each digest was expected to be 1 and the corresponding correction factors were used for normalization of the phosphorylated peptides. For the five non-phosphorylated peptides the ratio average is displayed with error bars as standard deviation. C) *In vitro* phosphorylation followed by partial trypsin digestion of recombinant Sir4N and indicated Sir4N phosphosite mutants. The tryptic peptides were separated by high resolution SDS-PAGE and analyzed by radiography. Peptide sequences to the right indicate the migration pattern of trypsin-digested *in vitro* phosphorylated standard peptides containing the indicated phospho-serine or -threonine residues D) Interaction of Sir4N with known interaction partners was analyzed by yeast two-hybrid analysis. Sir4N and Sir4N^GG^ or Sir4N^DD^ mutants were used as prey, for Yku80, Sir1 and Sif2 bait constructs that induce expression of the β-galactosidase gene upon interaction. At least three independent experiments were averaged for each value; data represent mean value ± s.e.m. E) Sir4N fragment indicated in Figure 7A and the respective mutants were expressed and purified from *E. coli*. DNA binding was performed and analyzed as in Figure 5A; data represent mean value ± s.e.m of three independent experiments. F) Sir4N fragments were bound to 6 mer arrays of nucleosomes and challenged with increasing amounts of MNase as in Figure 5D. Quantification of two independent experiments was used, data represent mean values.

To identify *in vivo* phosphoacceptor sites in Sir4, we first expressed a functional, epitope-tagged Sir4 from its endogenous locus. The Myc-tagged Sir4 protein was immunoprecipitated either from cycling cells or from cells that were arrested in G2/M phase by repressing *CDC20*, which encodes an essential anaphase-initiating factor (Figure S4A, S4B). We then used mass spectroscopy to identify the phosphorylated peptides. As predicted by Zill and colleagues [34], most of the phosphorylated amino acid residues were in the N-terminal half of the protein (Figure 7A and 7B, Figure S4C, Table 2). Moreover, ten of the twelve phosphorylated sites we identified contained the minimal consensus sequence for CDK, [S/T*]-P (in bold face in Figure 7A, Table 2), which can also be phosphorylated by the mitogen-activated protein kinase (MAPK). These sites were among those previously predicted to be targets for CDK and MAPK [71], [72].

**Table 2:** Summary of Sir4 phosphopeptides identified.

	peptide		p-site	Log	G2/M	SwissProt
K	KPV**pT**PNDKIPEREEK	S	22	x	x	
Y	SRPSTAIHT**pS**PHQPS	D	63	x	x	(62)
S	DVKPTSHKQLQQPK**p(SS)**PL	K	83/84		x	
R	SK**pT**SAGRIESNNPSHDASR	S	115		x	
L	TSKKIVP**pS**PKKVAI	D	342	x	x	yes
M	EILK**pS**PHLSKSPA	D	389	x	x	
K	SPHLSK**pS**PADRPQGR	R	395		x	
I	DSRNNTLNV**pT**PSKRPQLG	E	514	x	x	
S	DNFPV**p**(**S**L**S**)QPSKKSF	A	709/711	x	x	yes/yes
K	PSQIPTV**pS**PLGFEETK	L	932	x		
K	L**p**(**STT**)PTKSNRRVSH	S	942/943/944	x		
K	NVKPS**pS**PPDVK	S	1134	x	x	yes

Summary of Sir4 phosphopeptides identified in either cycling (Log) or G2/M arrested cells. Indicated are the identified peptides. The residues left and right indicate the cleavage sites and in bold is the phosphorylated residue within the peptide; the amino acid phosphorylated; the status of the cells in which it was detected; the presence of existing information in the SwissProt database. If the phosphorylation site is not uniquely identified, the potential site is in parenthesis. S/TP motifs are considered minimal consenses for either CDK or MAP kinase modification.

Five of these [S/T*]-P sites showed at least 1.5-fold higher levels of phosphorylation in G2/M as compared to cycling cells (Figure 7B). One of these was in the Sir4C domain (S1134), which, interestingly, is near a mapped SUMO-acceptor residue (K1128) within the Esc1-binding PAD domain [73]. However, mutation of this C-terminal phosphoacceptor site or the neighboring SUMO acceptor lysine yielded no detectable silencing- or anchoring-related phenotypes (data not shown). We therefore focused on the phosphoacceptor sites in the N-terminus of the protein. Within this domain, serine 63 (S63) was the site we detected most frequently over several experiments, while serine 84 (S84) showed a strict G2/M specificity.

To confirm the presence of CDK target sites within Sir4N, we exposed a recombinant Sir4N–GST fusion protein to a range of purified kinases *in vitro*. The recombinant protein was modified by CDK and protein kinase C, but not by yeast casein kinase II or the human MAP kinase, ERK (Figure S5A, S5B). To examine whether the sites modified by CDK *in vitro* corresponded to the sites phosphorylated *in vivo*, we mutated CDK consensus sites within the Sir4N domain (Figure 7A). We substituted consensus-site threonines 7 and 13 by alanines (A7 and A13), and serines 63 and 84 by glycine residues (Sir4N^GG^), alone and in combination (Figure 7C). The mutant Sir4N domains were purified and used as substrates for phosphorylation by CDK in the presence of γ^32^P-ATP. After trypsin digestion, the resulting phosphopeptides were resolved by high-resolution 1D gel electrophoresis (Figure 7C). The identities of the cleavage products were determined both by co-migration with synthesized, digested peptides and by the absence of signal in the mutants in which serine or threonine had been replaced by non-phosphoaccepting amino acids (Figure 7C and data not shown). Unlike the wild-type Sir4N protein, peptides carrying glycine substitutes at S63 and S84 lost almost all CDK-mediated phosphorylation *in vitro* (Figure 7C, Figure S5B). In contrast, alanine substitutions at T7 or T13 had only minor effects, alone or in combination. Given that S63 and S84 were phosphorylated by CDK both *in vitro* and in the endogenous protein recovered from mitotic cells, we propose that these two Sir4N residues are the major, physiological targets for CDK.

### Mutation of phosphoacceptor sites does not alter Sir4N interaction with Yku80, Sir1, Sif2, or DNA

To test the functional significance of Sir4N phosphorylation at S63 and S84 we analyzed the interactions of the non-phosphorylatable Sir4N mutant (Sir4N^GG^) and the mutant carrying a mutation that mimics the phosphoserine residues (Sir4N^DD^) with Sir1, Sif2 and Yku80. In yeast two-hybrid analyses, the interactions between Sir4N^GG^ or Sir4N^DD^ and Yku80, Sif2 or Sir1 were identical to the interactions between wild-type Sir4N and these binding partners (Figure 7D). Thus, at least in this assay, substitution of S63 and S84 by either G or D does not perturb the binding of known ligands to Sir4N.

We next tested mutant and wild-type Sir4N fragments for their ability to bind DNA and protect linker DNA from MNase digestion. Intriguingly, the Sir4N^GG^ mutant showed a higher affinity for DNA than the wild-type protein or the Sir4N^DD^ mutant, suggesting that Sir4N phosphorylation might weaken its interaction with DNA (Figure 7E).The incubation of Sir4N with CDK *in vitro* prior to DNA binding, increased the affinities of both wild-type Sir4N and the non-phosphorylatable Sir4N^GG^ mutant for DNA, due to nonspecific effects of the kinase (Figure S5C). Indeed, by performing MNase digestion of nucleosomes bound by Sir4N^GG^, Sir4N^DD^, or the wild-type protein, we found equal protection of linker DNAs in all cases (Figure 7F). We conclude that point mutations at these two major CDK phosphoacceptor sites in Sir4N do not substantially alter the affinity of the domain for either chromatin or DNA.

### Mutation of Sir4N phosphorylation sites affects the stability of gene repression *in vivo*

Despite the absence of *in vitro* phenotypes for Sir4N bearing mutated S63 and S84 residues, we checked the effects of these two phosphoacceptor site mutations on silencing *in vivo*. To test this, we introduced the double mutations S63G–S84G (*sir4^GG^*) or S63D–S84D (*sir4^DD^*) into the endogenous *SIR4* gene in a strain carrying both Tel5R::*ADE2* and Tel7L::*URA3* telomeric reporter genes. In the course of these experiments we serendipitously created an additional mutation at the Sir4 N-terminus, namely a proline to alanine substitution at residue 2 (*sir4^P2A^*). This substitution does not prevent cleavage of the initiator methionine or acetylation of the alanine at position 2 in contrast to the proline, and protein half life is predicted to be the same for either variant [74]. Indeed, we scored no significant effects on the half-life of Sir4 due to any of the mutations described above or below (data not shown).

**Figure 8:**
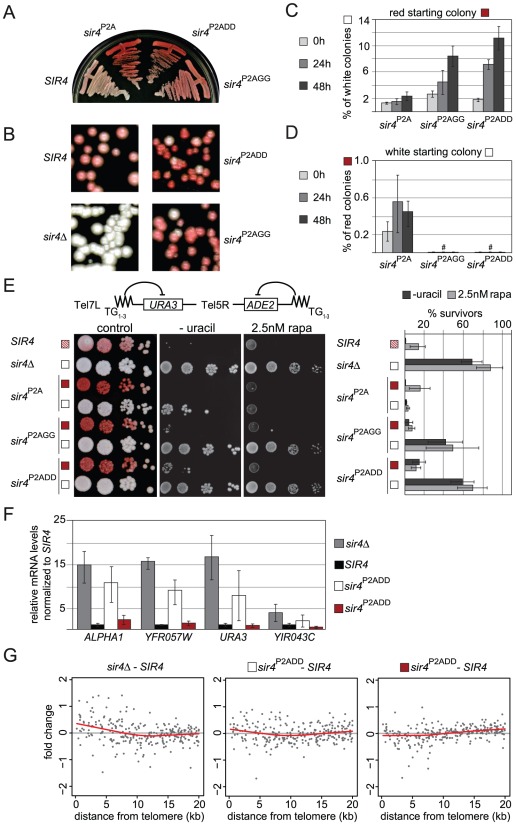
Sir4N phosphoacceptor site mutants show increased accumulation of active states and overall derepression of TPE. A, B) Single colony streaks for *ADE2* color assay monitor silencing of the indicated mutant strains (GA6018, GA5887, GA5888, GA5822, GA503). C,D) Quantification of cells swapping from a silent red to a de-repressed white state or vice versa. Single colonies were grown for the indicated time and dilutions plated on YPAD to monitor colony color. E) Single white or red colonies as in Figure 8C/8D were grown overnight and then spotted in dilution series onto YPAD plates for *ADE2* color development, or on plates lacking uracil. To test rapamycin sensitivity, cells were additionally spotted onto SC plates containing 2.5 nM rapamycin. Colony growth was quantified as % of survivors growing on plates lacking uracil or containing 2.5 nM rapamycin. Results are plotted in the bar graph (two independent isogenic strains, each scored in 4–8 experiments; combined data are represented as mean value ± s.e.m). We note that the *sir4^P2A^* effect is stronger at Tel5R than at Tel7L, possible because the silencing of reporters at Tel5R is much weaker to begin with [99], [100]. F) Relative quantification of mRNA of *HML-APLHA1* and the three indicated subtelomeric genes (see Figure 3C) in white or red colonies recovered from *SIR4*, *sir4*Δ and *sir4^P2ADD^* red and white colonies (GA503, GA5822, GA5887). Data from three biological replicates are represented as mean values ± sem G) Microarray analysis of *SIR4*, *sir4*Δ and *sir4^P2ADD^* red and white colonies (GA503, GA5822, GA5887). Plotted are the zero centered fold changes of log2 expression values of genes as a function of their distance from the telomere in relation to data for an isogenic *SIR4^+^* strain. Black spots represent single genes, the red line is lowess smoothed over all genes.

We first tested the effects of the background P2A mutation alone on *ADE2* silencing at Tel5R. We found that most *sir4^P2A^* colonies were darker red than wild-type colonies (Figure 8A), which indicates a more stable repression of *ADE2*. On the other hand, some colonies were completely white, indicating a low frequency of stable Tel5R::*ADE2* reporter derepression (Figure 8C–8E). This suggested to us that the P2A mutation stabilizes either “off” or “on” epigenetic states at Tel5R. When the P2A mutation was combined with the non-phosphorylatable *sir4^P2AGG^* mutation or the phospho-mimicking mutation (*sir4^P2ADD^*), we scored the same dark red color, but also noted that white colonies appeared at higher frequency (Figure 8A, 8B). To quantify this phenomenon, we cultured single white or dark red colonies from each strain in liquid culture and plated them out after 24 h and 48 h to score the status of the *ADE2* reporter by red *vs* white colony color. Whereas less than 1% of the *sir4^P2A^* colonies switched from red (*ADE2* repressed) to white (*ADE2* derepressed), we found that 6–10% of the *sir4^P2AGG^* or *sir4^P2ADD^* colonies switched color, indicating that these mutations render the repressed state less stable (Figure 8C). Conversely, we found that 0.4–0.6% of *sir4^P2A^* colonies switched from a derepressed to a repressed state (white to red), while the *sir4^P2AGG^* or *sir4^P2ADD^* strains remained completely derepressed, with no red colonies detected after 24 h of culturing of a white colony (Figure 8D). This argues that mutation of S63 and S84 generally destabilizes silencing or impairs re-establishment of a repressed state. The effects are particularly noticeable in combination with the *sir4^P2A^* mutation, which alone, for unknown reasons, stabilizes either state.

To test whether the effect of these mutations at Tel5R:*ADE2* held true for another telomere, we spotted overnight cultures of single colonies onto uracil-deficient plates, to score for expression of the Tel7L::*URA3* reporter gene (Figure 8E). We observed loss of *URA3* gene silencing in the *sir4^P2AGG^* and *sir4^P2ADD^* mutants. Moreover, the colonies growing on uracil-deficient plates were all white (*ADE2* derepressed), indicating that Tel7L and Tel5R reporter genes were derepressed simultaneously (Figure 8E). The loss of Tel7L::*URA3* repression was far more pronounced for the white colonies of *sir4^P2AGG^* and *sir4^P2ADD^* strains than for the *sir4^P2A^* mutation alone, arguing that alteration of the phosphorylation sites does indeed enhance derepression.

We next investigated whether the Sir4 phospho-site mutants caused general disruption of telomeric silencing when this is scored by growth on rapamycin (Figure 3A). Indeed, the white (derepressed) colonies of the *sir4^P2ADD^* and *sir4^P2AGG^* strains showed more resistance to rapamycin than the red (repressed) colonies, and were almost as resistant as the *sir4*Δ strain (Figure 8E). Quantification showed that this effect was far more pronounced in the *sir4^P2ADD^* and *sir4^P2AGG^* strains, than in the *sir4^P2A^* strain (15% for *sir4^P2A^*, 49% for *sir4^P2AGG^* and 69% for *sir4^P2ADD^*). As in the switching assay, the phospho-mimicking mutation *sir4^P2ADD^* produced the strongest derepressed state and strongest rapamycin resistance (Figure 8E).

We generalized this observation by scoring mRNA levels at native subtelomeric genes. This was done both by mRNA analysis for genes at two natural telomeres, Tel6R and Tel9R (Figure 3C) and by whole genome tiling arrays, that compared gene expression in wild-type *SIR4* cells with either red or white colonies of the *sir4^P2ADD^* mutant. The data in Figure 8F confirmed derepression at both natural telomeric genes and at Tel7L::*URA3* in white *sir4^P2ADD^* colonies, although not to the degree detected in the *sir4*Δ stain (Figure 8F). We also observed derepression of *HML* in the white *sir4^P2ADD^* cells. On the other hand, when we examine expression in the red *sir4^P2ADD^* colonies, we find that subtelomeric genes are as stably repressed in the red *sir4^P2ADD^* mutant cells as they are in a *SIR4* wild-type strain (Figure 8F). The microarray data confirmed this trend for subtelomeric genes (Figure 8G). By overlaying the transcriptional effects on all genes as a function of their distance from the telomere (red line is lowess smoothed over all genes), we score an increase in expression from genes within the first 5 kb from the telomere in both the *sir4*Δ and the white *sir4^P2ADD^* strains (Figure 8G). We also observed a generally stronger repressed state in the *sir4^P2ADD^* red colony for the genes that lie closest to telomeres, as expected. Thus the effects of the mutations on the *ADE2* reporter can be confirmed and extended to native subtelomeric genes.

In summary, thanks to the stabilizing effect of the *sir4^P2A^* background, we are able to demonstrate a defect in the *sir4^P2ADD^* and the *sir4^P2AGG^* strains in silencing. The derepressed state suggested by white colony color coincided with general derepression of TPE and with resistance to rapamycin, as reported for the *sir4*Δ strain. We note that the phenotypes were somewhat stronger for the phospho-mimicking *sir4^P2ADD^* mutant than for *sir4^P2AGG^*. Taken together, these results indicate that the Sir4 N-terminus helps modulate stable gene repression at telomeres by being phosphorylated on the target sites we have identified.

## Discussion

SIR-mediated transcriptional repression in budding yeast has been studied genetically and biochemically for over 20 years, yet we still do not fully understand the functions of its core components nor how it is regulated either during the cell cycle or in response to stress. In this study we addressed the molecular role and regulation of Sir4, the largest and least conserved Sir protein. On the basis of our new findings and a large body of earlier work, we can assign four roles to different domains of Sir4 and describe their functions in SIR-mediated repression. First, as described previously, the C-terminal half of the Sir4 protein has a scaffolding function that is essential for assembling Sir2 and Sir3 into the SIR complex and delivering it to chromatin (by binding to Rap1 and Yku). Second, the N-terminal 270 residues of Sir4 have a recruitment function by binding Sir1 and Yku80. Third, the Sir4N contributes to the tight association of the SIR complex with DNA *in vitro* and enhances nucleosomal linker protection. This domain is essential *in vivo* for repression of *HM* loci under suboptimal conditions (i.e. when the silencers in *HMR* are compromised or when Yku70 is absent) and contributes significantly to TPE. Finally, we find that both the extreme N-terminus and the adjacent central domain of Sir4 are heavily phosphorylated *in vivo* and that the mutation of two phosphoacceptor sites in the N-terminus affects the stability of subtelomeric repression. Given that Sir4 residues S63 and S84 are phosphorylated in mitotic cells, we speculate that the phosphorylation of Sir4N regulates the stability of TPE through the cell cycle.

### The C-terminus of Sir4 is sufficient to establish a silent chromatin structure

Marshall and colleagues proposed in 1987 [51] that both the N- and C-terminal domains of Sir4 were required for silencing of *HML*. By using a slightly shorter and significantly more stable C-terminal domain of Sir4 than that used by Marshall and colleagues (residues 747–1358), we show that the Sir4 N-terminus is dispensable for repression of intact *HML* and *HMR* loci, although not for the repression of subtelomeric reporter genes. The ability of Sir4C to silence *HM* reflects, in part, the strong redundancy in Sir factor recruitment pathways at *HM* loci, i.e. several silencer factors redundantly recruit Sir3 and Sir4 [20].

Consistent with our finding that Sir4C is sufficient to silence intact *HMR* in the presence of Sir2 and Sir3, we find that recombinant complexes containing Sir2, Sir3, and Sir4C are stable upon isolation, retain full histone H4K16 deacetylation activity and bind nucleosomal arrays *in vitro*. H4K16 deacetylation by Sir2 has been proposed to provoke a conformational change in the SIR complex that increases its binding to chromatin [28], [52], [75]. We do not know whether in the SIR complex the N-terminal domain of Sir4 contributes to this change in conformation, although we note that Sir4C-containing complexes retain deacetylation activity.

We explain the inability of a longer Sir4C fragment to repress (as described in Marshall *et al.*, 1987) by its instability (Figure S1B). Indeed, this is consistent with an earlier study of the *san1*-1 mutant, which partially restores mating in a strain expressing only a Sir4 C-terminal domain [76]. San1 is a ubiquitin ligase that targets misfolded proteins for degradation by the proteasome, and one of its targets is Sir4 [77]. Our work and this study suggest that a sufficiently stable Sir4C fragment provides all the essential interactions necessary for formation of silent chromatin at *HM* loci – most crucially a tight association with Sir2 and Sir3 to create an active, heterotrimeric complex that can interact stably with chromatin [13], [52]. It is not, however, sufficient for TPE.

### The Sir4 N-terminus enhances Sir4 binding to *HM* loci via protein- and DNA-interactions

Although the Sir4 C-terminus is sufficient for *HM* repression under certain conditions, we also found conditions that render the N-terminus essential for efficient *HM* silencing, namely when one of the recruiting elements was deleted at the *HMR-E* silencer (ΔA Orc1-Sir1 site or ΔB Abf1 site), or in the absence of *YKU70*. This weakens the recruitment of Sir3 or Sir4 to the *HM* silencers. The requirement for Sir4N under these conditions is consistent with its ability to bind Sir1 and Yku80. Sir1 recruits Sir4 to *HM* silencers by direct interaction with Orc1 [21]. The Yku70/80 complex can stabilizes silent chromatin by two means, first by recruiting the *HM* loci to Sir-clusters at the periphery [46], [49]and second by helping form a promoter-silencer interaction at *HM* loci through a looping mechanism [78]–[80]. Nevertheless, Yku70/80 is only essential for mating in the absence of Sir1 and vice versa [56], [57]. Consistent with this, we observe that Sir4N enhances mating efficiency in the absence of Yku70.

These two interactions, however, are probably not the only functions of the Sir4 N-terminus in *HM* silencing. First, there are two other sites of contact between Sir4 and the Yku70/80 complex [39], [42], and second, expression of a Sir4N-C fusion protein represses an *HMR* locus that lacks one of its binding sites for ORC (*HMR-E*ΔA; Figure 2D). This indicates a function for the Sir4 N-terminus that is independent of its interaction with Sir1. We propose that the additional function is its strong non-specific affinity for DNA, which contributes to a tighter interaction of the SIR complex to chromatin and to enhanced linker DNA protection *in vitro*.

### Full-length Sir4 is necessary for full subtelomeric repression

At telomeres, the Sir4 N-terminus is required for TPE. Moreover, while the expression of a Sir4N-C fusion slightly reduces mRNA levels compared to Sir4C, it could not suppress reporter genes when their promoters were induced. Since Sir1 is not required for subtelomeric repression, these effects are independent of Sir1 [15]. Rather, we suggest that the DNA-binding affinity of Sir4N increases binding of the SIR complex to telomeres to enhance the stability of repression. Full TPE, however, appears to require not only the first 270 amino acids of Sir4, but also the unstructured region between 270 and 744. Thus we suggest that another, yet unidentified function may be attributed to this domain.

Besides promoting tight association of the SIR holocomplex with DNA, the N-terminus of Sir4 may also regulate the strength or character of the Sir3–Sir4 interaction. We find that expression of Sir4C enhances the formation Sir3–EGFP foci even when there is no TPE. Similarly, Sir3-EGFP foci in the absence of TPE were observed in a strain overexpressing a non-acetylatable form of Sir3 [62], indicating that Sir protein clustering does not always lead to gene repression. It is possible that the Sir4C protein has a stronger affinity for Sir3 than does full-length Sir4. This is consistent with an earlier hypothesis that the Sir4 N-terminus interferes the binding of Sir3 to Sir4 [43], [81]. If true, the expression of Sir4C alone may lead to the sequestration of Sir3 into foci that antagonize repression. Consistent with this, we note that telomeres are highly sensitive to changes in Sir protein levels [31], and that Sir4C expression is somewhat toxic to cells, while that of the Sir4N–C fusion is not (Figure S2E and data not shown).

### The N-terminus is the major site of Sir4 phosphorylation and fine-tunes subtelomeric stress-response genes

In this study we characterize Sir4 as a phosphoprotein and map key phosphoacceptor sites in the N-terminal domain of the protein. We show that two sites that are phosphorylated by CDK *in vitro* are also phosphorylated in mitotic cells *in vivo*. Zill and colleagues [34] speculated that the N-terminus of Sir4 may be specialized for fine-tuning or regulating silencing in response to environmental factors. Indeed, we find that the N-terminus of Sir4 is its major site of phosphorylation *in vivo*.

What functions of Sir4 might be affected by its phosphorylation? As Sir4N is dispensable for *HM* silencing, but is essential for TPE, we reasoned that the state of Sir4 phosphorylation might affect the repression of subtelomeric genes, many of which are activated only under conditions of nutrient stress [59]. Phosphorylation of the Sir4N terminus by CDK may also destabilize silent chromatin in mitosis. Consistent with this, previous work has shown that SIR complexes are partially released from telomeres in mitotic yeast nuclei [63], [64], and yeast heterochromatin is most accessible to transcription factors and gene activation during G2/M phase [82]. Moreover, passage through mitosis, which is accompanied by CDK-dependent protein phosphorylation and dephosphorylation, is important for the establishment of silencing [83]–[85]. Although it is not clear why, we note that the partial release of factors from chromatin during mitosis is a common feature of eukaryotes. Heterochromatin protein 1 (HP1; [86]) and the Polycomb complex (PcG; [87]) are both partially released during mitosis in *Drosophila*. The Polycomb protein EZH2 is a direct target of CDK [88], [89], and HP1 release appears to be due to phosphorylation of histone H3 on Ser10 by Aurora B kinase [90], [91].

In yeast, we show that the substitution of the Sir4 phosphoacceptor sites S63 and S84 by acidic amino acids, or by non-phospho-accepting glycines, had only minor effects on the TPE (Figure S6). However, by combining these mutations with a fortuitous mutation of residue 2 (proline to alanine or P2A), we could observe that they indeed tend to derepress TPE. The *sir4^P2A^* mutation alone had a strong stabilizing effect on either silent or open epigenetic states, in that *ADE2* repression at Tel5R was seen to be either enhanced or abolished. The phospho-acceptor site mutants (*sir4^P2ADD^* or *sir4^P2AGG^*) has a tendency to derepress TPE and thereafter to retain the derepressed state, which is readily visible thanks to the stabilizing effect of *sir4^P2A^*. Indeed, re-establishing a repressed state occurred less frequently in the *sir4^P2ADD^* or *sir4^P2AGG^* mutant cells. Importantly, we then showed by microarray analysis that this was accompanied by a general derepression of subtelomeric genes, similar - albeit less pronounced - to that observed in *sir4*Δ cells. Together our analysis suggests that the modification of Sir4N S63 and S84 CDK target sites either directly derepresses TPE or interferes in the re-establishment of a repressed state. We suggest that the Sir4 N-terminal domain regulates repression both during the cell cycle and in response to environmental stress, which is most likely mediated by a MAP kinase cascade. Given the abundance of confirmed CDK phosphoacceptor sites in this domain, and putative MAP kinase sites, this may be one of the main functions of Sir4 N-terminus.

## Materials and Methods

### Plasmids, strains, and yeast methods

All strains and plasmids are described in Text S1. Standard techniques were used for cloning, yeast strain generation and growth. To obtain Sir4 expression similar to the endogenous levels, the Sir4 locus (1 kb 5′ of start and 250 bp 3′) was cloned into a CEN-ARS plasmid. Introduction of a *Nco*I site at the Sir4 start codon allowed subcloning of shorter Sir4 fragments and introduced the P2A mutation. To introduce the Sir4 phosphosite mutations into the genome, the respective plasmids were digested with *Sal*I and *Sac*I, purified fragments were integrated into a *sir4::KanMX6* (GA5822) strain. Positive clones were selected for growth on SC medium+0.1% 5-FOA and checked by sequencing of the genomic locus. At least two independent transformants were analyzed.

To check Sir4 expression levels, the CEN-ARS plasmids were transformed into a protease-deleted strain (GA73) and cells were grown to OD_600_<1 prior to lysis by bead-beating. Standard techniques were used for SDS-PAGE and immunoblotting. Mcm2 (yN-19) antibody is from Santa Cruz, anti Myc antibody 9E10. Sir2 and Sir4 antibody have been described previously [7], [92]. Yeast two-hybrid analysis was carried out as described previously but using the GA181 strain [93], [94].

### Silencing assays

For quantitative mating assays, plasmid transformed strains and the tester strain were grown overnight in SC medium. 10^7^ cells of the mating-tester strain (GA858) were mixed in 1 ml YPAD medium containing 2×10^6^ cells transformed with a given Sir4 plasmids and grown for 5 h at 30°C (see also [51]). Cells were then grown 3 days at 30°C on SD medium to select for diploids and SC medium – tryptophan/-methionine to normalize cell numbers.

Silencing of indicated reporter genes was performed as described [95], after growth overnight in selective media. Ten-fold dilution series starting at 10^7^ cells/ml were performed in triplicates on appropriate media. Silencing of the *ADE2* reporter was scored after 3 days growth on YPAD 30°C and subsequent maintenance at 4°C for 4 days.

### Recombinant protein purification

Sir4C-expressing baculovirus was generated using the BaculoGold linearized cDNA (BD Bioscience) and Cellfectin reagent (Invitrogen) according to manufacturer's instructions. SIR complexes were co-expressed in insect sf21 cells and purified as described previously [28], [52]. Buffers contained 10 mM TEA pH8 when the proteins were purified for gradient sedimentation. For gradient sedimentation, 200 µl Calmodulin-column eluate was layered on a 4 ml 5–25% glycerol gradient (10 mM TEA pH8, 150 mM sodium chloride, 0.01% Tween-20) prepared using a Gradient Master (BioComp) in Beckmann 11×61 polyallomer tubes. Gradients were centrifuged for 18 h at 4°C and 30'000 g and fractionated into 100 ul aliquots Samples of 20 µl were analyzed by SDS-PAGE and SyproRuby staining. Sir4N fragments were expressed in *E. coli* and purified from inclusion bodies using standard Ni-NTA techniques as described previously [52].

### *In vitro* phosphorylation and phosphopeptide mapping

Recombinant Sir4N-GST was purified from *E. coli* using standard procedures in PBS buffer. For phosphorylation assays, the proteins were incubated with the indicated kinases for 1 h at 37°C (a kind gift of E. Nigg; CDK2 (NM_001798 Proqinase)) and P^32^-γ-ATP. To analyze phosphorylation, Sir4N was run on a SDS-PAGE and proteins were detected by Coomassie staining and radiography. For phospho-peptide detection, Sir4N-GST was first digested by partial tryptic digest after *in vitro* phosphorylation. Peptides were lyophilized and analyzed on alkaline peptide gels and radiography according to the method of West and Bonner [96]. Briefly, samples were resuspended in loading buffer containing 0.125 M Tris-HCl pH 6.8 and 6 M urea. Peptides were then separated on 0.5 mm thick gels containing, 40% acrylamide, 0.037% bis-acrylamide, 0.75 M Tris-HCl pH 8.8. Gels were run at 10 mA for approximately 4 h. Peptide size was determined by co-migration with synthesized peptides that were phosphorylated and loaded alongside.

### Chromatin and DNA binding, MNase digestion

Chromatin and DNA binding as well as MNase digestions were performed as described previously [28], [52]. Briefly, 6 mer of nucleosomes diluted to 2.5×10^−8^ M were incubated with increasing amounts of indicated proteins in 10 mM TEA pH7.4, 25 mM sodium chloride for 20 min on ice and analyzed on 0.7% native agarose gels (0.2× TB) run at 4°C. DNA binding assays were performed using a 167 bp 601-Widom sequence DNA fragment Cy5-labeled by Klenow enzyme at an *Ava*I site. DNA bindings were performed in 150 mM sodium chloride at a DNA concentration of 2.5×10^−9^ M, using the same conditions as for chromatin binding. Linker DNA protection assays were performed by adding 0.25–1 U MNase and 1 mM calcium chloride to 1 pmol chromatin that had been pre-incubated with indicated amounts of Sir proteins. After 10 min on ice 5 mM EGTA was added and samples were deproteinized by incubation with proteinase K for 30 min at 30°C. Deacetylation, expression and purification of homogeneously H4K16 reactions were carried out as described previously [28]. 10 nM of purified Sir2–Sir3–Sir4 and Sir2–Sir3–Sir4C complex were incubated with 70 nM of H4K16^ac^ histone octamers with or without 150 µM of NAD. The reaction was carried out at 30°C in 50 mM Tris pH 8, 50 mM sodium chloride, 2.7 mM potassium chloride, 1 mM magnesium chloride and 0.005% Tween-20. The reaction was stopped at the indicated time points by addition of Laemmli sample buffer and samples were analyzed by 4–12% SDS PAGE by immuno blotting using H4K16^ac^ antibody (Millipore 07-329) and H3 antibody (Abcam ab1791).

### Immunoprecipitation and phosphosite mapping

Cells (GA5691, GA5589, GA1275) were grown overnight to OD_600_ = 0.6 in YPA-galactose media, then shifted to YPA-glucose for 2 h at 30°C for mitotic arrest (G2/M cells). Cycling cells were grown in the same carbon source, but did not contain the GALp:CDC20 allele that leads to G2/M arrest in glucose. Cells were harvested, washed once in ice-cold PBS and resuspended in one pellet volume of lysis buffer without detergent (50 mM HEPES pH7.5, 500 mM sodium acetate, 5 mM magnesium acetate, 0.1 mM EDTA, 5% glycerol [32]). The resuspended cells were snap frozen in liquid nitrogen and broken using a ball-mill (3×3 min at 30 1/s; Retsch MM4000); the cell powder was stored at −80°C. For immunoprecipitations, the cell powder was mixed with an equal volume of lysis buffer containing protease and phosphatase inhibitors and 1% Triton X-100. After thawing on ice for 5 min, cell extract was cleared by centrifugation and 5 mg of proteins were incubated at 4°C with 50 µl of Affi-prep protein A beads (BioRad) crosslinked to 9E10 antibodies [97]. Beads were washed with lysis buffer and stably bound proteins were eluted twice with 1.5× bead volume of 2 M glycine pH2 which was neutralized afterwards by Tris pH 8.0. For mass spectroscopy analysis, the eluates were processed by reduction and alkylation of the cysteines followed by sequential digestion with AspN and chymotrypsin or with trypsin only. The peptides were separated by nano-HPLC (Agilent 1100 nanoLC system, Agilent Technologies) coupled to an LTQ Orbitrap Velos hybrid mass spectrometer (Thermo Scientific) operated in positive mode using a top 5 DDA method. Inclusion lists were partially added to the method to search for expected peptides and to confirm already identified phosphorylated Sir4 peptides. Phosphorylated peptides and phosphosites were determined searching SwissProt data base restricted to *S. cerevisiae* using Mascot 2.3 (Matrix Science). Resulting sequences were inspected manually. Relative quantification was performed by integration of LC–MS extracted ion chromatograms. The peak areas of the corresponding phosphorylated peptides were normalized to the average of the peak areas of five non-phosphorylated Sir4 peptides. For prediction of CDK and MAPK sites, GPS2.1 software was used [72].

### Microscopy

C-terminally EGFP-tagged Sir3 (GA3128, GA6287, GA6288) was monitored in live cells grown to mid-log phase in SC medium and then embedded in an agarose pad as described [98]. For quantification of Sir3-EGFP foci, all images were taken the same day and treated with the same threshold to quantify foci versus intense foci (above that threshold).

### mRNA purification, QPCR, and microarray

Cells of indicated strains/colony color were grown to OD_600_>0.6 and mRNA was purified using Qiagen mini RNeasy Kit. Reverse Transcriptase (RT) reaction was performed using ProtoScript AMV Kit (NEB#E6550). For QPCR, 0.5 µl of the RT reaction was used in a total volume of 10 µl using the GoTaq qPCR Master Mix (Promega, A6002), sybr green method and the ONE STEP fast cycler (ABI). For primers see Text S1. Values were normalized to *ACT1* to account for samples differences and then to Sir4.

For microarrays, 100 ng of total RNA were amplified with the GeneChip WT Double-Stranded Target Assay (Affymetrix) and hybridized to GeneChip *S. cerevisiae* Tiling 1.0R Arrays following the “GeneChip Whole Transcript (WT) Double-Stranded Target Labeling Assay Manual” (Affymetrix) with a hybridization time of 16 h. The Affymetrix Fluidics protocol FS450_0001 was used for washing. Scanning was performed with Affymetrix GCC Scan Control v. 3.0.0.1214 on a GeneChip Scanner 3000 with autoloader. Raw data CEL files were read into R (version 2.14.1) using the Bioconductor (version 2.9) package Affy and a custom CDF package (available upon request). Probe sets were summarized and probe set-level values normalized with the RMA function. Gene coordinates for *S. cerevisiae* genes (EF3) were downloaded from Biomart (central.biomart.org) and chromosome length information was retrieved from the chromInfo table of the UCSC genome browser (genom.ucsc.edu) for SacCer_Apr2011/sacCer3. Fold changes were calculated using the lmFit and eBayes functions as implemented in the limma package. Fold changes for telomeric genes were centered around zero and plotted against the distance to the closest chromosome end. Smoothing was performed with the lowess (locally weighted scatterplot smoothing) function and fold changes for each contrast were scaled and centered using the function scale.

## Supporting Information

Figure S1 An additional 16amino acids renders the Sir4C fragment labile and unable to silence at *HMR*. A) Plasmids expressing Sir4C (residues 747–1358), a slightly longer Sir4 C-terminal fragment (residues 731–1358) or full length Sir4, under the Sir4 promoter and terminator, were transformed into yeast strains carrying a *TRP1* reporter at *HMR* and the indicated genotype at the *SIR4* locus (GA5886, *sir4*Δ; GA6072, *sir4N* (1–270)). Ten-fold dilution series were grown on plates selective for the plasmid (control) with or without tryptophan, to score for *HMR* repression. B) The plasmids indicated in A) were transformed into a strain lacking major vacuolar proteases and the *SIR4* gene (GA73). Extracts of logarithmically growing cells were analyzed by immunoblotting using a Sir4 and Mcm2 antibody. Asterisks indicate Sir4 bands.
(EPS)

Figure S2 Sir4C and Sir4N-C mediate mating type but not telomeric repression even in the absence of Rif1. A) Mating of cells expressing the Sir4N-C fusion. A Sir4 deletion strain (GA5822) was transformed with the indicated Sir4 plasmids and a ten-fold dilution series thereof was mixed in YPAD with a mating tester strain GA858. After incubation at 30°C, cells were spotted on plates lacking tryptophan for growth and on SD plates to score for mating. All Sir4 constructs support mating in the *sir4*Δ strain. Using quantitative mating assays as in Figure 1C, we note that Sir4N-C cells mate less efficiently than Sir4C-expressing cells (efficiency is 0.73%±0.11% compared to wild type Sir4), although they repress the *HMR::TRP1* reporter more efficiently. We think that our mating assay overestimates the efficiency of mating of Sir4C cells, because the growth rate of these cells is much slower than wild-type or Sir4N-C-expressing cells (i.e. see Figure 3A for slower growth and Figure 6B for distorted nuclei). This gives the resulting diploids (now *SIR4*/*SIR4C*) a growth advantage over single colony haploids (*SIR4C*), which are used to normalize mating efficiency. B) Silencing of URA3 reporter at Tel7L as in Figure 3A, just that a strain background expressing the first 270 residues of Sir4 (sir4N; GA5809) was used. C) To monitor the *ADE2* reporter at Tel5R, the transformed cells (as in A) were spotted in ten-fold dilution series onto YPAD plates, and color developed after cell growth at 4°C. The reddish color indicates repression, which is manifest only in the wild-type strain (*SIR4*) or in *sir4*Δ complemented with a plasmid expressing full-length *SIR4*. D) Mating assay as in A) using strains as in C) to test silencing at *HML* of *rif1*Δ strains. All constructs except the vector control support *HM* repression and allow efficient mating. E) Telomeric silencing was tested in *rif1*Δ strains carrying both *URA3* and *ADE2* reporters as indicated, by ten-fold dilution series on plates lacking uracil or on YPAD media, respectively (GA503, GA7137, GA7144). F) Sir4 ChIP. Sir4, Sir4C and Sir4N-C were expressed in a *sir4*Δ (GA5822) strain and their binding to *HML* and telomeres analyzed by ChIP/QPCR using an antibody raised against Sir4C. Primers used for QPCR are in Supplementary Information S1. Bars represent averages of biological duplicates/QPCR triplicates, data represent mean value ± s.e.m. To the left are schemes of the *HML* loci and telomeres analyzed indicating the location of QPCR primer pairs (short black lines).
(EPS)

Figure S3 Sir4C repression is not altered in *sir4*Δ cells containing Sir3-EGFP however Sir4C slightly de-represses in cells *SIR4* expressing Sir3-EGFP. The indicated Sir4 plasmids were transformed into a yeast strain carrying Tel7L::*URA3* and an EGFP tag on the endogenous Sir3 and the indicated genotype at the endogenous *SIR4* locus (*SIR4*, GA3128; *sir4N*, GA6287; *sir4*Δ, GA6288). Ten-fold dilution series were grown on selective plates for the indicated plasmids (control), and the expression of the *URA3* reporter was scored on plates lacking uracil or containing 0.1% 5-FOA. Sir4C expression derepresses slightly in a wild-type *SIR4* background. For mating, the cells were mixed with a 10× excess of mater tester strain (GA858) on YPAD plates, grown overnight at 30°C and then replica plated on plates selecting for diploids.
(EPS)

Figure S4 Analysis of immunopurified Sir4 and MS spectrum of pS63. A, B) Immunopurified Sir4-Myc was separated on 4–12% NuPAGEs Novex Bis-Tris Gels and analyzed by silver staining or immunoblotting using antibodies against Myc and Sir2. A strain with untagged Sir4 was used as a specificity control (GA5589, GA5691). The full-length Sir4 band in A) is indicated with an asterisk. IN: cell extract, input of IP; P: pellet of whole cell extract; SN: supernatant after IP; IP: glycine eluates from beads. C) pS63 can be detected. CID spectrum of the Sir4 peptide SRPSTAIHTpSPHQPS (*m/z* 841.88) derived from a combined AspN and chymotrypsin digest. The neutral loss of phosphoric acid is indicated by ‘-98’, the loss of water by ‘-18’.
(EPS)

Figure S5 *In vitro* phosphorylation of Sir4N by CDK does not influence DNA binding. A) *In vitro* phosphorylation of recombinant Sir4N with human Cyclin dependent kinase (CDK), Protein Kinase C (PKC), Casein kinase II (CKII) and Ets regulated kinase (ERK). For Sir4N, radiography and Coomassie staining are shown. For the control substrates H1, Casein and MBP (Myelin Basic Protein) only the radiography data is shown below the respective Sir4N lane. B) *In vitro* phosphorylation of Sir4N, Sir4N^DD^ or H1 by CDK2/cycA kinase, shows that much CDK phosphorylation is lost when S63/S84 are mutated. C) DNA binding assay of Sir4N and Sir4N^GG^ using the same conditions as in B) but without radioactive labeling. Three independent experiments were quantified, data represent mean values ± sem.
(EPS)

Figure S6 *sir4^DD^* or *sir4^GG^* mutations silence efficiently at telomeres. Sir4 phosphoacceptor site mutants as in Figure 8A/8B but without the P2A mutation were integrated at the endogenous *SIR4* locus into a strain carrying both the Tel5R*::ADE2* reporter gene and Tel7L*::URA3* reporter gene. A colony of each strain was grown overnight and spotted onto YPAD for color visualization of the *ADE2* reporter in ten-fold dilution series. Strains used are GA503, GA5822, GA6362, and GA6363.
(EPS)

Text S1 The Text S1 file contains supplementary methods (ChIP) and tables for yeasts strains, plasmids and QPCR primers used in this study.
(DOCX)

## References

[1] Braunstein M, Rose AB, Holmes SG, Allis CD, Broach JR (1993). Transcriptional silencing in yeast is associated with reduced nucleosome acetylation.. Genes Dev.

[2] Suka N, Suka Y, Carmen AA, Wu J, Grunstein M (2001). Highly specific antibodies determine histone acetylation site usage in yeast heterochromatin and euchromatin.. Mol Cell.

[3] Loo S, Rine J (1994). Silencers and domains of generalized repression.. Science.

[4] Gottschling DE (1992). Telomere-proximal DNA in Saccharomyces cerevisiae is refractory to methyltransferase activity in vivo.. Proc Natl Acad Sci U S A.

[5] Singh J, Klar AJ (1992). Active genes in budding yeast display enhanced in vivo accessibility to foreign DNA methylases: a novel in vivo probe for chromatin structure of yeast.. Genes Dev.

[6] Raghuraman MK, Brewer BJ, Fangman WL (1997). Cell cycle-dependent establishment of a late replication program.. Science.

[7] Gotta M, Laroche T, Formenton A, Maillet L, Scherthan H (1996). The clustering of telomeres and colocalization with Rap1, Sir3, and Sir4 proteins in wild-type Saccharomyces cerevisiae.. J Cell Biol.

[8] Ottaviani A, Gilson E, Magdinier F (2008). Telomeric position effect: from the yeast paradigm to human pathologies?. Biochimie.

[9] Rusche LN, Kirchmaier AL, Rine J (2003). The establishment, inheritance, and function of silenced chromatin in Saccharomyces cerevisiae.. Annu Rev Biochem.

[10] Gasser SM, Cockell MM (2001). The molecular biology of the SIR proteins.. Gene.

[11] Moazed D (2001). Common themes in mechanisms of gene silencing.. Mol Cell.

[12] Buhler M, Gasser SM (2009). Silent chromatin at the middle and ends: lessons from yeasts.. Embo J.

[13] Cubizolles F, Martino F, Perrod S, Gasser SM (2006). A homotrimer-heterotrimer switch in Sir2 structure differentiates rDNA and telomeric silencing.. Mol Cell.

[14] Rine J, Herskowitz I (1987). Four genes responsible for a position effect on expression from HML and HMR in Saccharomyces cerevisiae.. Genetics.

[15] Aparicio OM, Billington BL, Gottschling DE (1991). Modifiers of position effect are shared between telomeric and silent mating-type loci in S. cerevisiae.. Cell.

[16] Shore D, Stillman DJ, Brand AH, Nasmyth KA (1987). Identification of silencer binding proteins from yeast: possible roles in SIR control and DNA replication.. Embo J.

[17] Buchman AR, Lue NF, Kornberg RD (1988). Connections between transcriptional activators, silencers, and telomeres as revealed by functional analysis of a yeast DNA-binding protein.. Mol Cell Biol.

[18] Moretti P, Freeman K, Coodly L, Shore D (1994). Evidence that a complex of SIR proteins interacts with the silencer and telomere-binding protein RAP1.. Genes Dev.

[19] Sussel L, Shore D (1991). Separation of transcriptional activation and silencing functions of the RAP1-encoded repressor/activator protein 1: isolation of viable mutants affecting both silencing and telomere length.. Proc Natl Acad Sci U S A.

[20] Brand AH, Micklem G, Nasmyth K (1987). A yeast silencer contains sequences that can promote autonomous plasmid replication and transcriptional activation.. Cell.

[21] Triolo T, Sternglanz R (1996). Role of interactions between the origin recognition complex and SIR1 in transcriptional silencing.. Nature.

[22] Tanner KG, Landry J, Sternglanz R, Denu JM (2000). Silent information regulator 2 family of NAD- dependent histone/protein deacetylases generates a unique product, 1-O-acetyl-ADP-ribose.. Proc Natl Acad Sci U S A.

[23] Imai S, Armstrong CM, Kaeberlein M, Guarente L (2000). Transcriptional silencing and longevity protein Sir2 is an NAD-dependent histone deacetylase.. Nature.

[24] Armstrong CM, Kaeberlein M, Imai SI, Guarente L (2002). Mutations in Saccharomyces cerevisiae gene SIR2 can have differential effects on in vivo silencing phenotypes and in vitro histone deacetylation activity.. Mol Biol Cell.

[25] Hecht A, Laroche T, Strahl-Bolsinger S, Gasser SM, Grunstein M (1995). Histone H3 and H4 N-termini interact with SIR3 and SIR4 proteins: a molecular model for the formation of heterochromatin in yeast.. Cell.

[26] Georgel PT, Palacios DeBeer MA, Pietz G, Fox CA, Hansen JC (2001). Sir3-dependent assembly of supramolecular chromatin structures in vitro.. Proc Natl Acad Sci U S A.

[27] Johnson A, Li G, Sikorski TW, Buratowski S, Woodcock CL (2009). Reconstitution of heterochromatin-dependent transcriptional gene silencing.. Mol Cell.

[28] Oppikofer M, Kueng S, Martino F, Soeroes S, Hancock S (2011). The dual role of H4K16 acetylation in the establishment of yeast silent chromatin.. Embo J.

[29] Strahl-Bolsinger S, Hecht A, Luo K, Grunstein M (1997). SIR2 and SIR4 interactions differ in core and extended telomeric heterochromatin in yeast.. Genes Dev.

[30] Rusche LN, Kirchmaier AL, Rine J (2002). Ordered nucleation and spreading of silenced chromatin in Saccharomyces cerevisiae.. Mol Biol Cell.

[31] Cockell M, Gotta M, Palladino F, Martin SG, Gasser SM (1998). Targeting Sir proteins to sites of action: a general mechanism for regulated repression.. Cold Spring Harb Symp Quant Biol.

[32] Rudner AD, Hall BE, Ellenberger T, Moazed D (2005). A nonhistone protein-protein interaction required for assembly of the SIR complex and silent chromatin.. Mol Cell Biol.

[33] Ehrentraut S, Hassler M, Oppikofer M, Kueng S, Weber JM (2011). Structural basis for the role of the Sir3 AAA^+^ domain in silencing: Interaction with Sir4 and unmethylated histone H3K79.. Genes & Development.

[34] Zill OA, Scannell D, Teytelman L, Rine J (2010). Co-evolution of transcriptional silencing proteins and the DNA elements specifying their assembly.. PLoS Biol.

[35] Tanny JC, Kirkpatrick DS, Gerber SA, Gygi SP, Moazed D (2004). Budding yeast silencing complexes and regulation of Sir2 activity by protein-protein interactions.. Mol Cell Biol.

[36] Chang JF, Hall BE, Tanny JC, Moazed D, Filman D (2003). Structure of the coiled-coil dimerization motif of Sir4 and its interaction with Sir3.. Structure.

[37] Murphy GA, Spedale EJ, Powell ST, Pillus L, Schultz SC (2003). The Sir4 C-terminal coiled coil is required for telomeric and mating type silencing in Saccharomyces cerevisiae.. J Mol Biol.

[38] Moretti P, Shore D (2001). Multiple interactions in Sir protein recruitment by Rap1p at silencers and telomeres in yeast.. Mol Cell Biol.

[39] Tsukamoto Y, Kato J, Ikeda H (1997). Silencing factors participate in DNA repair and recombination in Saccharomyces cerevisiae.. Nature.

[40] Laroche T, Martin SG, Gotta M, Gorham HC, Pryde FE (1998). Mutation of yeast Ku genes disrupts the subnuclear organization of telomeres.. Curr Biol.

[41] Mishra K, Shore D (1999). Yeast Ku protein plays a direct role in telomeric silencing and counteracts inhibition by rif proteins.. Curr Biol.

[42] Taddei A, Hediger F, Neumann FR, Bauer C, Gasser SM (2004). Separation of silencing from perinuclear anchoring functions in yeast Ku80, Sir4 and Esc1 proteins.. Embo J.

[43] Roy R, Meier B, McAinsh AD, Feldmann HM, Jackson SP (2004). Separation-of-function mutants of yeast Ku80 reveal a Yku80p-Sir4p interaction involved in telomeric silencing.. J Biol Chem.

[44] Andrulis ED, Neiman AM, Zappulla DC, Sternglanz R (1998). Perinuclear localization of chromatin facilitates transcriptional silencing.. Nature.

[45] Maillet L, Boscheron C, Gotta M, Marcand S, Gilson E (1996). Evidence for silencing compartments within the yeast nucleus: a role for telomere proximity and Sir protein concentration in silencer-mediated repression.. Genes Dev.

[46] Hediger F, Neumann FR, Van Houwe G, Dubrana K, Gasser SM (2002). Live imaging of telomeres: yKu and Sir proteins define redundant telomere-anchoring pathways in yeast.. Curr Biol.

[47] Andrulis ED, Zappulla DC, Ansari A, Perrod S, Laiosa CV (2002). Esc1, a nuclear periphery protein required for Sir4-based plasmid anchoring and partitioning.. Mol Cell Biol.

[48] Taddei A, Gasser SM (2004). Multiple pathways for telomere tethering: functional implications of subnuclear position for heterochromatin formation.. Biochim Biophys Acta.

[49] Gartenberg MR, Neumann FR, Laroche T, Blaszczyk M, Gasser SM (2004). Sir-mediated repression can occur independently of chromosomal and subnuclear contexts.. Cell.

[50] Taddei A, Van Houwe G, Nagai S, Erb I, van Nimwegen E (2009). The functional importance of telomere clustering: global changes in gene expression result from SIR factor dispersion.. Genome Res.

[51] Marshall M, Mahoney D, Rose A, Hicks JB, Broach JR (1987). Functional domains of SIR4, a gene required for position effect regulation in Saccharomyces cerevisiae.. Mol Cell Biol.

[52] Martino F, Kueng S, Robinson P, Tsai-Pflugfelder M, van Leeuwen F (2009). Reconstitution of yeast silent chromatin: multiple contact sites and O-AADPR binding load SIR complexes onto nucleosomes in vitro.. Mol Cell.

[53] Cockell M, Renauld H, Watt P, Gasser SM (1998). Sif2p interacts with Sir4p amino-terminal domain and antagonizes telomeric silencing in yeast.. Curr Biol.

[54] Pijnappel WW, Schaft D, Roguev A, Shevchenko A, Tekotte H (2001). The S. cerevisiae SET3 complex includes two histone deacetylases, Hos2 and Hst1, and is a meiotic-specific repressor of the sporulation gene program.. Genes Dev.

[55] Pillus L, Rine J (1989). Epigenetic inheritance of transcriptional states in S. cerevisiae.. Cell.

[56] Patterson EE, Fox CA (2008). The Ku complex in silencing the cryptic mating-type loci of Saccharomyces cerevisiae.. Genetics.

[57] Vandre CL, Kamakaka RT, Rivier DH (2008). The DNA end-binding protein Ku regulates silencing at the internal HML and HMR loci in Saccharomyces cerevisiae.. Genetics.

[58] Renauld H, Aparicio OM, Zierath PD, Billington BL, Chhablani SK (1993). Silent domains are assembled continuously from the telomere and are defined by promoter distance and strength, and by SIR3 dosage.. Genes Dev.

[59] Ai W, Bertram PG, Tsang CK, Chan TF, Zheng XF (2002). Regulation of subtelomeric silencing during stress response.. Mol Cell.

[60] Hardy CF, Sussel L, Shore D (1992). A RAP1-interacting protein involved in transcriptional silencing and telomere length regulation.. Genes Dev.

[61] Lowary PT, Widom J (1998). New DNA sequence rules for high affinity binding to histone octamer and sequence-directed nucleosome positioning.. J Mol Biol.

[62] Ruault M, De Meyer A, Loiodice I, Taddei A (2011). Clustering heterochromatin: Sir3 promotes telomere clustering independently of silencing in yeast.. J Cell Biol.

[63] Laroche T, Martin SG, Tsai-Pflugfelder M, Gasser SM (2000). The dynamics of yeast telomeres and silencing proteins through the cell cycle.. J Struct Biol.

[64] Smith CD, Smith DL, DeRisi JL, Blackburn EH (2003). Telomeric protein distributions and remodeling through the cell cycle in Saccharomyces cerevisiae.. Mol Biol Cell.

[65] Martin SG, Laroche T, Suka N, Grunstein M, Gasser SM (1999). Relocalization of telomeric Ku and SIR proteins in response to DNA strand breaks in yeast.. Cell.

[66] Mills KD, Sinclair DA, Guarente L (1999). MEC1-dependent redistribution of the Sir3 silencing protein from telomeres to DNA double-strand breaks.. Cell.

[67] Radman-Livaja M, Ruben G, Weiner A, Friedman N, Kamakaka R (2011). Dynamics of Sir3 spreading in budding yeast: secondary recruitment sites and euchromatic localization.. Embo J.

[68] Ray A, Hector RE, Roy N, Song JH, Berkner KL (2003). Sir3p phosphorylation by the Slt2p pathway effects redistribution of silencing function and shortened lifespan.. Nat Genet.

[69] Holt LJ, Tuch BB, Villen J, Johnson AD, Gygi SP (2009). Global analysis of Cdk1 substrate phosphorylation sites provides insights into evolution.. Science.

[70] Ubersax JA, Woodbury EL, Quang PN, Paraz M, Blethrow JD (2003). Targets of the cyclin-dependent kinase Cdk1.. Nature.

[71] Miller ML, Jensen LJ, Diella F, Jorgensen C, Tinti M (2008). Linear motif atlas for phosphorylation-dependent signaling.. Sci Signal.

[72] Xue Y, Ren J, Gao X, Jin C, Wen L (2008). GPS 2.0, a tool to predict kinase-specific phosphorylation sites in hierarchy.. Mol Cell Proteomics.

[73] Denison C, Rudner AD, Gerber SA, Bakalarski CE, Moazed D (2005). A proteomic strategy for gaining insights into protein sumoylation in yeast.. Mol Cell Proteomics.

[74] Hwang CS, Shemorry A, Varshavsky A (2011). N-terminal acetylation of cellular proteins creates specific degradation signals.. Science.

[75] Liou GG, Tanny JC, Kruger RG, Walz T, Moazed D (2005). Assembly of the SIR complex and its regulation by O-acetyl-ADP-ribose, a product of NAD-dependent histone deacetylation.. Cell.

[76] Schnell R, D'Ari L, Foss M, Goodman D, Rine J (1989). Genetic and molecular characterization of suppressors of SIR4 mutations in Saccharomyces cerevisiae.. Genetics.

[77] Dasgupta A, Ramsey KL, Smith JS, Auble DT (2004). Sir Antagonist 1 (San1) is a ubiquitin ligase.. J Biol Chem.

[78] Valenzuela L, Dhillon N, Dubey RN, Gartenberg MR, Kamakaka RT (2008). Long-range communication between the silencers of HMR.. Mol Cell Biol.

[79] Hofmann JF, Laroche T, Brand AH, Gasser SM (1989). RAP-1 factor is necessary for DNA loop formation in vitro at the silent mating type locus HML.. Cell.

[80] Weiss K, Simpson RT (1998). High-resolution structural analysis of chromatin at specific loci: Saccharomyces cerevisiae silent mating type locus HMLalpha.. Mol Cell Biol.

[81] Moazed D, Kistler A, Axelrod A, Rine J, Johnson AD (1997). Silent information regulator protein complexes in Saccharomyces cerevisiae: a SIR2/SIR4 complex and evidence for a regulatory domain in SIR4 that inhibits its interaction with SIR3.. Proc Natl Acad Sci U S A.

[82] Aparicio OM, Gottschling DE (1994). Overcoming telomeric silencing: a trans-activator competes to establish gene expression in a cell cycle-dependent way.. Genes Dev.

[83] Kirchmaier AL, Rine J (2006). Cell cycle requirements in assembling silent chromatin in Saccharomyces cerevisiae.. Mol Cell Biol.

[84] Miller AM, Nasmyth KA (1984). Role of DNA replication in the repression of silent mating type loci in yeast.. Nature.

[85] Martins-Taylor K, Dula ML, Holmes SG (2004). Heterochromatin spreading at yeast telomeres occurs in M phase.. Genetics.

[86] Kellum R, Raff JW, Alberts BM (1995). Heterochromatin protein 1 distribution during development and during the cell cycle in Drosophila embryos.. J Cell Sci.

[87] Buchenau P, Hodgson J, Strutt H, Arndt-Jovin DJ (1998). The distribution of polycomb-group proteins during cell division and development in Drosophila embryos: impact on models for silencing.. J Cell Biol.

[88] Wei Y, Chen YH, Li LY, Lang J, Yeh SP (2011). CDK1-dependent phosphorylation of EZH2 suppresses methylation of H3K27 and promotes osteogenic differentiation of human mesenchymal stem cells.. Nat Cell Biol.

[89] Chen S, Bohrer LR, Rai AN, Pan Y, Gan L (2010). Cyclin-dependent kinases regulate epigenetic gene silencing through phosphorylation of EZH2.. Nat Cell Biol.

[90] Fischle W, Tseng BS, Dormann HL, Ueberheide BM, Garcia BA (2005). Regulation of HP1-chromatin binding by histone H3 methylation and phosphorylation.. Nature.

[91] Hirota T, Lipp JJ, Toh BH, Peters JM (2005). Histone H3 serine 10 phosphorylation by Aurora B causes HP1 dissociation from heterochromatin.. Nature.

[92] Perrod S, Cockell MM, Laroche T, Renauld H, Ducrest AL (2001). A cytosolic NAD-dependent deacetylase, Hst2p, can modulate nucleolar and telomeric silencing in yeast.. Embo J.

[93] Golemis EA, Serebriiskii I, Finley RL, Kolonin MG, Gyuris J (2001). Interaction trap/two-hybrid system to identify interacting proteins.. Curr Protoc Protein Sci.

[94] Bjergbaek L, Cobb JA, Tsai-Pflugfelder M, Gasser SM (2005). Mechanistically distinct roles for Sgs1p in checkpoint activation and replication fork maintenance.. Embo J.

[95] Gotta M, Palladino F, Gasser SM (1998). Functional characterization of the N terminus of Sir3p.. Mol Cell Biol.

[96] Pantazis P, West MH, Bonner WM (1984). Phosphorylation of histones in cells treated with hypertonic and acidic media.. Mol Cell Biol.

[97] Herzog F, Peters JM (2005). Large-scale purification of the vertebrate anaphase-promoting complex/cyclosome.. Methods Enzymol.

[98] Meister P, Gehlen LR, Varela E, Kalck V, Gasser SM (2010). Visualizing yeast chromosomes and nuclear architecture.. Methods Enzymol.

[99] Gottschling DE, Aparicio OM, Billington BL, Zakian VA (1990). Position effect at S. cerevisiae telomeres: reversible repression of Pol II transcription.. Cell.

[100] Mondoux MA, Zakian VA (2007). Subtelomeric elements influence but do not determine silencing levels at Saccharomyces cerevisiae telomeres.. Genetics.

