# Supplementary material for: Regulating Repression: Roles for the Sir4 N-Terminus in Linker DNA Protection and Stabilization of Epigenetic States
Source: PLoS Genet. 2012 May 24;8(5):e1002727. doi: 10.1371/journal.pgen.1002727 (PMC3359979; doi:10.1371/journal.pgen.1002727)
Supplement: Text S1 — The Text S1 file contains supplementary methods (ChIP) and tables for yeasts strains, plasmids and QPCR primers used in this study. (DOCX) [file pgen.1002727.s007.docx]

**Supplementary materials and methods**

**Sir4 ChIP**

ChIP was carried out as described previously [1], with the adjustments that Sir4 antibody [2] was used at 2ug/20 μl beads and that DNA was purified using the AccuPrep® PCR purification kit (Bioneer) after overnight incubation at 65°C to reverse the crosslinking. Each 10 μl QPCR reaction used 0.5 μl of the eluate as described in the main Materials and Methods section. Enrichment was normalized to the *ACT1* locus.

**Table S1.** Yeast strains used in this study.

| strain | Genotype | source |
| --- | --- | --- |
| GA181  W303alpha | *MATalpha; ade2-1 trp1-1 his3-11 his3-15 ura3-1 leu2-3 leu2-112, can1-100* |  |
| YPH499 (S288C) | *MATalpha; ura3-52, lys2-801, ade2-101, trp1del63, his3del200, leu2del1* |  |
| GA73 | (S288C) *prb1-1122 pep4-3 prc1-407 prb1-1122 leu2::sir4* | [3] |
| GA1275 | YPH499*; MATalpha SIR4-13myc::kanMX6* |  |
| GA5589 | YPH499*; MATa cdc20::LEU2 GAL_CDC20::TRP1* | this study |
| GA5691 | YPH499*; MATa cdc20::LEU2 GAL_CDC20::TRP1 SIR4-13myc::kanMX6* | this study |
| GA484 | W303*; MATalpha; hmr::TRP1* | [4] |
| GA485 | W303*; MATalpha; hmrΔB::TRP1* | [4] |
| GA486 | W303*; MATalpha; hmrΔE::TRP1* | [4] |
| GA5886 | GA484*; sir4::kanMX6* | this study |
| GA6072 | GA484*; sir4-1-270::ADH1Term::kanMX6* | this study |
| GA6888 | GA485 *sir4::kanMX6* | this study |
| GA6889 | GA485*; sir4-1-270::ADH1Term::kanMX6* | this study |
| GA6890 | GA486*; sir4::kanMX6* | this study |
| GA6891 | GA486*; sir4-1-270::ADH1Term::kanMX6* | this study |
| GA858 | W303*; MATalpha; hom3 canR* |  |
| GA503 | YPH499*; MATa ppr1::HIS3 adh4::URA3-TEL 7L 5R::ADE2-TEL* | [5] |
| GA5822 | GA503*; sir4::kanMX6* | this study |
| GA5809 | GA503*; sir4-1-270::ADH1term::kanMX6* | this study |
| GA6062 | GA503*; sir1::cNAT* | this study |
| GA6063 | GA503*; sir1::cNAT sir4-1-270::ADH1term::kanMX6* | this study |
| GA6064 | GA503*; sir1::cNAT sir4::kanMX6* | this study |
| GA6069 | GA503*; ku70::cNAT* | this study |
| GA6070 | GA503*; ku70::cNAT sir4-1-270::ADH1term::kanMX6* | this study |
| GA6071 | GA503*; ku70::cNAT sir4::kanMX6* | this study |
| GA7137 | GA503; *sir4::kanMX6*  *rif1::cNAT* | this study |
| GA7144 | GA503; *rif1::cNAT* | this study |
| GA6018 | GA503*; sir4::SIR4-P2A* | this study |
| GA6462 | GA503*; sir4::SIR4-P2A* | this study |
| GA6362 | GA503*; sir4::SIR4-GG* | this study |
| GA6363 | GA503*; sir4::SIR4-DD* | this study |
| GA5888 | GA503*; sir4::SIR4-P2AGG* | this study |
| GA5901 | GA503*; sir4::SIR4-P2AGG* | this study |
| GA5887 | GA503*; sir4::SIR4-P2ADD* | this study |
| GA6017 | GA503*; sir4::SIR4-P2ADD* | this study |
| GA3128 | GA503*; Sir3-EGFP::kanMX6* | this study |
| GA6287 | GA3128*;sir4-1-270::ADH1term::TRP1* | this study |
| GA6288 | GA3128*; sir4::TRP1* | this study |
| GA-1461 | W303; *Nup49:GFP his::lacI-GFP::HIS* ARS607::lacO | [6] |

**Table S2.** Plasmids used in this study.

| name | number | insert | backbone | source |
| --- | --- | --- | --- | --- |
| pRST-*SIR4*-P2A | 2460 | Sir4-P2A 5'/3' Sir4 locus | pRS414 | this study |
| pRST-*SIR4*-P2ADD | 2461 | Sir4-DDP2A 5'/3' Sir4 locus | pRS414 | this study |
| pRST-*SIR4*-P2AGG | 2463 | Sir4-GGP2A 5'/3' Sir4 locus | pRS414 | this study |
| pRST-*SIR4* | 2740 | Sir4 5'/3' Sir4 locus | pRS414 | this study |
| pRST-*SIR4*-DD | 2741 | Sir4-DD 5'/3' Sir4 locus | pRS414 | this study |
| pRST-*SIR4*-GG | 2742 | Sir4-GG 5'/3' Sir4 locus | pRS414 | this study |
| pRST | 2605 | 5'/3' Sir4 locus NcoI/SalI | pRS414 | this study |
| pRST-*SIR4* 721-1358 | 2607 | Sir4C 721-1358 5'/3' Sir4 locus | pRS414 | this study |
| pRST-*SIR4C* | 2609 | Sir4C 747-1358 5'/3' Sir4 locus | pRS414 | this study |
| pRST-*SIR4N*-*C* | 2765 | Sir4N-HA-TEV-Sir4C 5'/3' Sir4 locus | pRS414 | this study |
| pRSH | 2716 | 5'/3' Sir4 locus NcoI/SalI | pRS413 | this study |
| pRSH-*SIR4* | 2717 | Sir4 5'/3' Sir4 locus | pRS413 | this study |
| pRSH-*SIR4C* | 2718 | Sir4C 747-1358 5'/3' Sir4 locus | pRS413 | this study |
| pRSH-*SIR4N-C* | 2963 | Sir4N-HA-TEV-Sir4C 5'/3' Sir4 locus | pRS413 | this study |
| pRSL | 2719 | 5'/3' Sir4 locus NcoI/SalI | pRS415 | this study |
| pRSL-*SIR4* | 2720 | Sir4 5'/3' Sir4 locus | pRS415 | this study |
| pRSL-*SIR4C* | 2721 | Sir4C 747-1358 5'/3' Sir4 locus | pRS415 | this study |
| pVL1392-*SIR4C*-CBP | 2051 | Sir4C 747-1358 | pVL1392 | this study |
| pSH18-34 | 359 | lexA_op(8x)_ – lacZ |  | [7] |
| pGAL-LexA | 965 | LexA_(1-202)_DNA-BD | pEG202 | [8] |
| pLexA-*SIR1C* | 2653 | Sir1 aa 344-654 | pGAL-LexA | this study |
| pLexA-*SIF2* | 2650 | Sif2 aa 1-535 | pGAL-LexA | this study |
| pLexA-*yKU80* | 2326 | yKu80 aa 1-630 | pAT4 | [9] |
| pJG4-5 | 363 | AD-B42 | pJG-45 |  |
| pB42-*SIR4N* | 2444 | Sir4N aa 1-270 | pJG-45 | this study |
| pB42-*SIR4N*-DD | 2497 | Sir4N-DD aa 1-270 | pJG-45 | this study |
| pB42-*SIRr4N*-GG | 2498 | Sir4N-GG aa 1-270 | pJG-45 | this study |
| pET-*SIR4N* | 2147 | Sir4N aa 2-272 | pET30a | [10] |
| pET-*SIR4N*-GG | 2641 | Sir4N-GG aa 2-272 | pET30a | this study |
| pET-*SIR4N*-DD | 2642 | Sir4N-DD aa 2-272 | pET30a | this study |
| pGEXkg-*SIR4N*-A13 | 616 | Sir4N-A13 | pGEX | this study |
| pGEXkg-*SIR4N*-A7 | 617 | Sir4N-A7 | pGEX | this study |
| pGEXkg-*SIR4N*-GG | 618 | Sir4N-GG | pGEX | this study |
| pGEXkg-*SIR4N*-A7/GG | 619 | Sir4N-A7-GG | pGEX | this study |

**Table S3.** Primers used for quantitative PCR

| Name | Sequence | gene | reference |
| --- | --- | --- | --- |
| SG5780 | CACAGTTTGGCTCCGGTGTA | *HMLα1* | [11] |
| SG5781 | CCGCGTGCCATTCTTCAG | *HMLα1* | [11] |
| SG418 | CTTGTATTAGACGAGGGACGGAGTG | *HML-E* | this study |
| SG419 | ACAGAGGGTCACAGCACTACTACAG | *HML-E* | this study |
| SG5783 | GGAATGATCTTGGAAATCGATCA | *YFR057W* | [11] |
| SG5784 | CTAGTGTCTATAGTAAGTGCTCGG | *YFR057W* | [12] |
| SG4791 | TTGACCCATACCGACCATGATA | *ACT1 rev* | [13] |
| SG5788 | AGGTTGCTGCTTTGGTTATTGA | *ACT1 fwd* | this study |
| SG5789 | AGCTGCGGTGTTTACAAGT | *YIR043C* | this study |
| SG5790 | ACTACCGGAAACAAGAAACGTG | *YIR043C* | this study |
| SG5861 | CCGCCAAGTACAATTTTTTAC | *URA3* | [14] |
| SG5862 | CAACCAATCGTAACCTTCATC | *URA3* | [14] |

**Supplemental material references**

1. Cubizolles F, Martino F, Perrod S, Gasser SM (2006) A homotrimer-heterotrimer switch in Sir2 structure differentiates rDNA and telomeric silencing. Mol Cell 21: 825-836.

2. Perrod S, Cockell MM, Laroche T, Renauld H, Ducrest AL, et al. (2001) A cytosolic NAD-dependent deacetylase, Hst2p, can modulate nucleolar and telomeric silencing in yeast. Embo J 20: 197-209.

3. Dubey DD, Davis LR, Greenfeder SA, Ong LY, Zhu JG, et al. (1991) Evidence suggesting that the ARS elements associated with silencers of the yeast mating-type locus HML do not function as chromosomal DNA replication origins. Mol Cell Biol 11: 5346-5355.

4. Sussel L, Shore D (1991) Separation of transcriptional activation and silencing functions of the RAP1-encoded repressor/activator protein 1: isolation of viable mutants affecting both silencing and telomere length. Proc Natl Acad Sci U S A 88: 7749-7753.

5. Gottschling DE (1992) Telomere-proximal DNA in Saccharomyces cerevisiae is refractory to methyltransferase activity in vivo. Proc Natl Acad Sci U S A 89: 4062-4065.

6. Taddei A, Hediger F, Neumann FR, Bauer C, Gasser SM (2004) Separation of silencing from perinuclear anchoring functions in yeast Ku80, Sir4 and Esc1 proteins. Embo J 23: 1301-1312.

7. Sato T, Hanada M, Bodrug S, Irie S, Iwama N, et al. (1994) Interactions among members of the Bcl-2 protein family analyzed with a yeast two-hybrid system. Proc Natl Acad Sci U S A 91: 9238-9242.

8. Bjergbaek L, Cobb JA, Tsai-Pflugfelder M, Gasser SM (2005) Mechanistically distinct roles for Sgs1p in checkpoint activation and replication fork maintenance. Embo J 24: 405-417.

9. Ferreira HC, Luke B, Schober H, Kalck V, Lingner J, et al. (2011) The PIAS homologue Siz2 regulates perinuclear telomere position and telomerase activity in budding yeast. Nat Cell Biol 13: 867-874.

10. Martino F, Kueng S, Robinson P, Tsai-Pflugfelder M, van Leeuwen F, et al. (2009) Reconstitution of yeast silent chromatin: multiple contact sites and O-AADPR binding load SIR complexes onto nucleosomes in vitro. Mol Cell 33: 323-334.

11. Yang B, Britton J, Kirchmaier AL (2008) Insights into the impact of histone acetylation and methylation on Sir protein recruitment, spreading, and silencing in Saccharomyces cerevisiae. J Mol Biol 381: 826-844.

12. Darst RP, Garcia SN, Koch MR, Pillus L (2008) Slx5 promotes transcriptional silencing and is required for robust growth in the absence of Sir2. Mol Cell Biol 28: 1361-1372.

13. Schawalder SB, Kabani M, Howald I, Choudhury U, Werner M, et al. (2004) Growth-regulated recruitment of the essential yeast ribosomal protein gene activator Ifh1. Nature 432: 1058-1061.

14. Martins-Taylor K, Dula ML, Holmes SG (2004) Heterochromatin spreading at yeast telomeres occurs in M phase. Genetics 168: 65-75.
